# Influence of Season on Biodegradation Rates in Rivers

**DOI:** 10.1021/acs.est.3c10541

**Published:** 2024-03-25

**Authors:** Run Tian, Malte Posselt, Luc T. Miaz, Kathrin Fenner, Michael S. McLachlan

**Affiliations:** † Department of Environmental Science (ACES), Stockholm University, Stockholm 10691, Sweden; ‡ Eawag, Swiss Federal Institute of Aquatic Science and Technology, Dübendorf 8600, Switzerland; § Department of Chemistry, University of Zürich, Zürich 8057, Switzerland

**Keywords:** biodegradation, seasonality, up- and downstream, micropollutants, total cell count

## Abstract

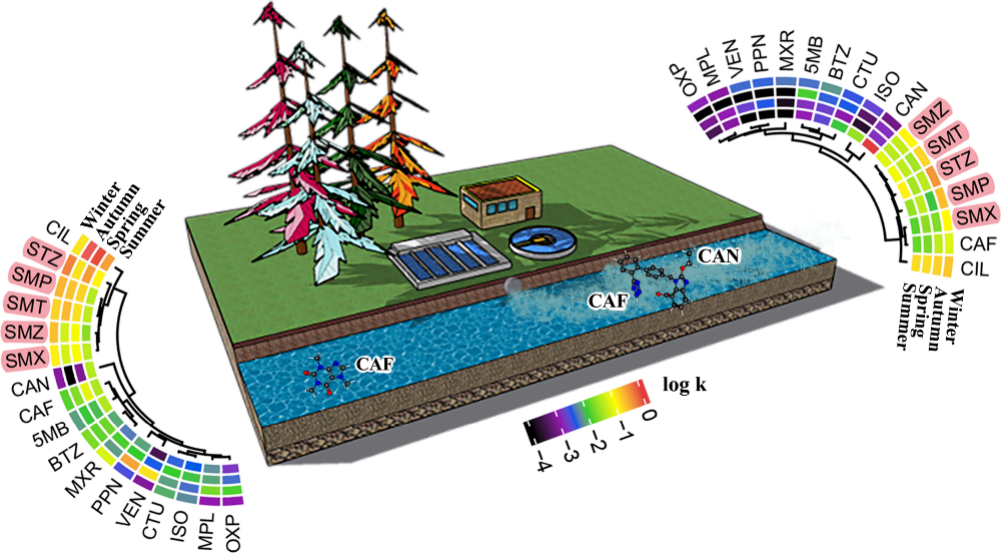

Biodegradation plays a key role in the fate of chemicals in the environment. The variability of biodegradation in time can cause uncertainty in evaluating the environmental persistence and risk of chemicals. However, the seasonality of biodegradation in rivers has not yet been the subject of environmentally relevant testing and systematic investigation for large numbers of chemicals. In this work, we studied the biodegradation of 96 compounds during four seasons at four locations (up- and downstream of WWTPs located on two Swedish rivers). Significant seasonality (ANOVA, *p* < 0.05) of the first-order rate constant for primary biodegradation was observed for most compounds. Variations in pH and total bacterial cell count were not the major factors explaining the seasonality of biodegradation. Deviation from the classical Arrhenius-type behavior was observed for most of the studied compounds, which calls into question the application of this relationship to correct biodegradation rate constants for differences in environmental temperature. Similarities in magnitude and seasonality of biodegradation rate constants were observed for some groups of chemicals possessing the same functional groups. Moreover, reduced seasonality of biodegradation was observed downstream of WWTPs, while biodegradation rates of most compounds were not significantly different between up- and downstream.

## Abstract 2

The environmental biodegradation rate of many chemicals varies seasonally, frequently in a manner that deviates from the Arrhenius relationship.

## Introduction

The reversibility of chemical exposure is an important indicator of our ability to manage future contamination problems.^1^ Biodegradation is often the most important process contributing to the reversibility of chemical exposure. Yet, in assessing chemical exposure, the rate of biodegradation is typically the largest source of uncertainty.^2^ There is evidence that biodegradation rates in the environment are temporally variable.^3−8^ For instance, in field studies investigating contaminant attenuation in Swedish and Swiss rivers, pronounced seasonality was found in the biodegradation of various pharmaceuticals.^4,6^ Although the seasonality of biodegradation rates can have a major impact on overall chemical fate, there has been no systematic investigation of the seasonal dependence of biodegradation rates in aquatic systems, i.e., for a large and diverse enough set of chemicals to draw generally valid conclusions on the extent of seasonality and major influencing factors.

Biodegradation in the natural environment is the result of a complex interplay of environmental factors, both directly and indirectly through their influence on the microbial community.^8−15^ pH is a parameter with a direct mechanistic link to biodegradation rates. Changes in pH will change the speciation (neutral fraction, f_N_) of ionizable chemicals, thereby changing their bioavailability.^16^ There is strong empirical evidence showing that the neutral species of a chemical crosses bacterial cell membranes much more readily than the charged species, and that, therefore, it is mostly the neutral fraction that is available for biodegradation.^17−19^ We note that speciation does not influence availability for degradation by extracellular enzymes,^16^ but we have little evidence that this is important for many organic water pollutants. Furthermore, there is empirical evidence indicating that changes in pH affect the biodegradation rate, and that this effect can be largely eliminated by normalizing the rate constant by the neutral fraction.^9,10^

To describe the variability of biodegradation rates, one conceptual approach is to describe the rate of chemical dissipation as the product of the chemical concentration, a second-order reaction rate constant, and a biodegradation capacity.^16^ The biodegradation capacity represents the equivalent amount of microbial biomass that is active in transforming the chemical at the rate defined by the second-order rate constant, and it can be varied over time to describe seasonality. The total microbial biomass has been assumed to be the most influential system-specific difference affecting aquatic biodegradation^20^ and has been suggested as a descriptor of the biodegradation capacity. Notably, this approach implies that the activity of the specific microorganisms degrading the chemicals scales with the total biomass. A number of proxies for total microbial biomass have been proposed such as total organic carbon, but bacterial cell density has been argued to be a more precise measure.^4^

Temperature has been reported to be another important factor influencing the biodegradation rates of chemicals.^21,22^ Enzyme-catalyzed reactions are believed to be the rate-limiting step in the biodegradation of most organic contaminants. Like for other chemical reactions, enzyme-catalyzed reaction rates are expected to be temperature-dependent in a manner described by the Arrhenius relationship, and hence, it has been surmised that the biodegradation rate shows an Arrhenius-type temperature dependence. This assumption has already been incorporated into regulatory practice; e.g., the European Chemicals Agency (ECHA) recommends using the Arrhenius equation to extrapolate first-order biodegradation rate constants or half-lives from one temperature to another.^23,24^ However, in the natural environment, the microbial community adapts to seasonal changes in environmental conditions,^11,25,26^ which could confound the thermodynamic effect of temperature.^5,8^ Indeed, several studies have reported faster biodegradation of a number of organic contaminants during seasons with lower temperatures.^5,8^ To date, the evidence for an Arrhenius type-dependence of k on temperature is thus conflicting and comparatively weak.

Additionally, anthropogenic contamination has been found to have an impact on microbial communities^13,27,28^ and the biodegradation capacity of aquatic systems.^29,30^ Wastewater discharge is one of the major sources of anthropogenic contamination in the environment. It has been shown to impact biodegradation downstream of the wastewater treatment plants (WWTPs), causing changes in the biodegradation kinetics of some chemicals compared to upstream.^30^ Previous studies have found seasonal variation in pollutant removal at WWTPs^31,32^ and in the occurrence of pollutants in effluent recipient waters.^33^ Such effects might be additional drivers of seasonality in biodegradation downstream of WWTPs.

Against this scientific background, this study aimed to quantify the seasonal variation of chemical biodegradation rates in rivers for a large number of chemicals and to explore different explanations for the observed seasonality. We conducted a series of modified OECD 309 experiments that were designed to maximally mirror field conditions by assessing the dissipation at the beginning of the incubation when the microbial community most closely resembled the community in the field.^34^ We used river water and sediment from four sites up- and downstream of two WWTPs located on two Swedish rivers to carry out biodegradation experiments during four seasons. We estimated the first-order rate constant for primary biodegradation (*k*) of 96 chemicals in those 16 experiments. We then used these data to investigate the seasonality of *k* and to what extent it could be explained by pH-induced changes in bioavailability, active biomass, as described using the total cell count (TCC), and temperature. In addition, we compared k between upstream and downstream of the WWTPs and investigated the influence of treated wastewater releases on the seasonality of biodegradation in the rivers.

## Materials and Methods

### Test Compounds

A mixture of 129 compounds was prepared for the biodegradation experiments. They were chosen based on their low log *D*_*OW*_ (80% of the compounds with a log *D*_*OW*_ < 3 at pH 7.4, Table S1) and their occurrence in treated wastewater and surface water. These compounds cover multiple use classes (pharmaceuticals, agrochemicals, cosmetics, food additives, and industrial chemicals) and multiple chemical classes, for several of which we expected similar initial biotransformation reaction pathways according to existing literature (sulfonamides, thioethers, acetanilides, phenylureas, amides, amines, etc.).^35−37^ Information on the standards and labeled internal standards used for analysis is provided in the Supporting Information (SI, S1).

### Biodegradation Experiments

Sampling was carried out up- and downstream of two wastewater treatment plants (Fors WWTP: FUp and FDown, Knivsta WWTP: KUp and KDown) located on two small Swedish rivers close to Stockholm (Vitsån and Knivstaån) during 4 seasons (winter: 2022.03.17, spring: 2022.06.08, summer: 2022.08.01, autumn: 2022.10.04). The upstream river sections received no wastewater input. The downstream sampling sites were approximately 500 to 700 m downriver of the WWTP outfalls to ensure complete mixing of effluent with river water. The effluent from Fors WWTP and Knivsta WWTP was diluted by the river flow by a factor of approximately 5 to 15 and 1 to 5, respectively, on the sampling days. The dilution factor was calculated as the ratio of the flow rate of the river to effluent. The flow rates of the rivers were obtained from the Swedish Meteorological and Hydrological Institute.^38^ The flow rates of the effluents were provided by the WWTPs. The sampling and experimental setup followed the protocol of an OECD 309 test with slight modifications that we have previously shown to provide better environmental relevance than the standard OECD 309 test.^34^ Briefly, samples of surface water and the top 3–5 cm layer of sediment were collected and transported in a cooled and insulated container from the sampling sites to the lab. 100 mL of water was frozen immediately at −20 °C for future analysis. The incubations began within 24 h of sampling. For the incubations, sediment was sieved to 2 mm, homogenized, and mixed with river water (50 g wet solid L^–1^) in sealed Erlenmeyer flask incubators. All experiments were carried out in the dark at the river water temperature. The experiment does not distinguish between the degradation by bacteria, algae, and other organisms, but there is evidence showing that degradation by bacteria dominates in aquatic systems^29^ and, furthermore, the activity of algae in our incubations was reduced by the dark conditions. All experiments for a given season were conducted simultaneously at the same temperature (winter: 4 °C, spring: 17 °C, summer: 19 °C, autumn: 11 °C). An orbital shaker was used to keep the sediment in suspension during the incubation. Total organic carbon (TOC) was measured in the sieved sediment.

Test treatments (3 replicates), sorption controls (SC, sterilized sediment-water mixtures for distinguishing biodegradation from sorption, 2 replicates), and a hydrolysis control (sterilized river water for distinguishing biodegradation and sorption from hydrolysis) were spiked with a 1 mL aqueous mixture of 129 compounds to a concentration of 1 μg L^–1^ each. Dissipation of the test compounds was monitored by analyzing subsamples taken from the water phase of each vessel after 0, 2, 5, 9, 18 h, 1, 2, 4, 6, 8, and 10 days. We turned off the orbital shaker 10 min before sampling the test vessels to allow the sediment to settle so that we sampled primarily the aqueous portion of the incubation mixture. The incubators were shaken for 10 min before the start of the experiment to ensure complete mixing. During incubation, water parameters (pH, temperature, and conductivity) were measured manually on each sampling day in each flask, and dissolved oxygen was measured on a daily basis. Further information on the characteristics of the sampled river sections and the experimental systems is given in sections S1.2 and S1.3 of the SI.

### Bacterial Total Cell Counts Quantification by Flow Cytometry

Total cell count (TCC) was used as a measure of active biomass in this study and calculated based on cell density measured in water and sediment samples. Measurements were conducted in the sieved and homogenized sediment and river water prior to filling the incubation flasks, from a flask at the start of the incubation and from the test flasks at the end of the incubation. Sampling and sample preparation for cell density analysis were based on a study by Seller et al.^39^ and are described in detail in section S1.2 of the SI. Briefly, 5 mL of water samples (or 5 g of wet sediment) were fixated with 5 mL buffer fixative (4% paraformaldehyde buffered with 0.1% pyrophosphate) in amber glass vials and stored at 4 °C until analysis. Sediment samples were ultrasonicated (4 × 20 s) and the supernatant solution containing detached cell suspension was transferred to 2 mL Eppendorf tubes for measurement. Water samples and the supernatant solution transferred from sediment samples were diluted, stained with 1% SYBR Green (1:100 dilution of 10 mM Tris buffer), and incubated at 37 °C for 15 min. Measurements were conducted on a BD Accuri C6 Flow Cytometer (BD, Belgium). The sum of the bacterial cell densities measured in the water and sediment samples prior to filling the incubation flasks was used to calculate the “field TCC” for each experiment. For information on the flow cytometry measurements, TCC calculation, and its variation during different periods of incubation, please see Section S2 of the SI.

### Chemical Analysis and Data Processing

Chemical analysis and data processing were carried out as described by Tian et al.^34^ using an ultrahigh-performance liquid chromatography system coupled to a Q Exactive HF Hybrid Quadrupole-Orbitrap mass spectrometer (UHPLC-Orbitrap-MS/MS, Thermo Fisher Scientific, San Jose, CA) with electrospray ionization (ESI). Data processing was carried out in Compound Discoverer 3.3. Filtered river water spiked with 1 μg L^–1^ of all of the studied compounds and 10 μg L^–1^ of the internal standards was used as a quality control sample (QC), which was measured every 6 subsamples. A 15-point matrix-matched calibration curve was measured with concentrations ranging from 1 ng L^–1^ to 10 μg L^–1^. The limit of detection (LOD) was calculated based on the lowest detected calibration standard. The lowest detected calibration standard within the linear range was set as the limit of quantification (LOQ). Out of the 129 spiked compounds, 33 could not be quantified in all seasonal test treatments; therefore, their biodegradation could not be assessed. About 60% of the unquantifiable compounds have log *D*_*OW*_ > 3 at pH 7.4, while only 8 out of 96 quantified compounds have log *D*_*OW*_ > 3 at pH 7.4 (Table S1). More information about the reasons for nonquantification is given in Table S3.

The evaluation of the kinetics was based on the peak area, as the variability in matrix effects between samples was low for most analytes (e.g., the median relative standard deviation of the peak area of the internal standards between samples from test treatments was 9%). Rate constants *k* were calculated using linear least-squares regression of the natural logarithm of the chemical’s peak area versus time from the start of the incubation as long as dissipation was first-order. In agreement with Tian et al.,^34^ only the initial *k* was considered environmentally relevant when biphasic kinetics were observed. For biphasic kinetics, the calculation of the biodegradation kinetic parameters and the recognition of the breakpoint was carried out using a Python algorithm that we developed based on the Chow test (*chowclassifier*, section S3.3 of the SI).^40,41^ All data from the 3 replicates were used simultaneously in the Python algorithm. The calculated *k* was considered valid when the 99% confidence interval did not cross 0 and was significantly different from 0 (*p* value <0.05). We also calculated *k* for each replicate using linear regression, and these data were used for assessing replicate precision and when the ANOVA test was applied. Before calculating *k*, the dissipation in the sorption controls during the experiment was subtracted from the dissipation in test treatments. The fraction of compound that was dissolved (*f*_Dis_) at *t* = 0 (i.e., 10 min after adding the standard mixture) was estimated as the quotient in peak area between the sorption control and the hydrolysis control, and the observed *k* (*k*_observed_) was divided by *f*_Dis_ to correct for the dissolved fraction (SI S5).
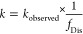


The concentration of studied compounds in nonspiked river water samples was determined using the matrix-matched calibration curve. More information on quality control and quality assurance is provided in section S3 of the SI. Dissipation in the sorption controls and the consequences for the estimation of *k* are detailed in sections S4 and S5 of the SI.

## Results and Discussion

### Large Seasonal Variation in Biodegradation Rate Constant

All experiments for the 4 different seasons mirrored the environmental conditions well (temperature, pH, DO, and conductivity). DO in the water phase always increased to the saturated level (90% to 100%) within the first 5 h of incubation and then became stable. All of the environmental parameters were stable during incubation and reproducible between the 3 replicates (section S3.1, SI).

All of the 96 compounds were biodegraded in at least one seasonal incubation (Supplemental Data set S1). Notably, only 16 compounds exhibited significant sorption to the sediment prior to the commencement of the experiment (higher than 2-fold difference in peak area between hydrolysis control and sorption controls). This was consistent across seasonal experiments at different locations (section S4, SI). All of the 16 compounds are bases. Chemical dissipation during incubation in sorption controls was small compared to dissipation in the test treatments for 94% of the *k* estimations (i.e., *k* derived from the sorption controls was at least 2.5 times smaller than *k* derived from the test treatments). The relative standard deviation of *k* between the 3 replicates was <30% for 77% of the degraded chemicals. The results supported the environmental relevance and precision of the measurements, and they agreed with our previous evaluation of the modified OECD 309 test.^34^

**Figure 1:**
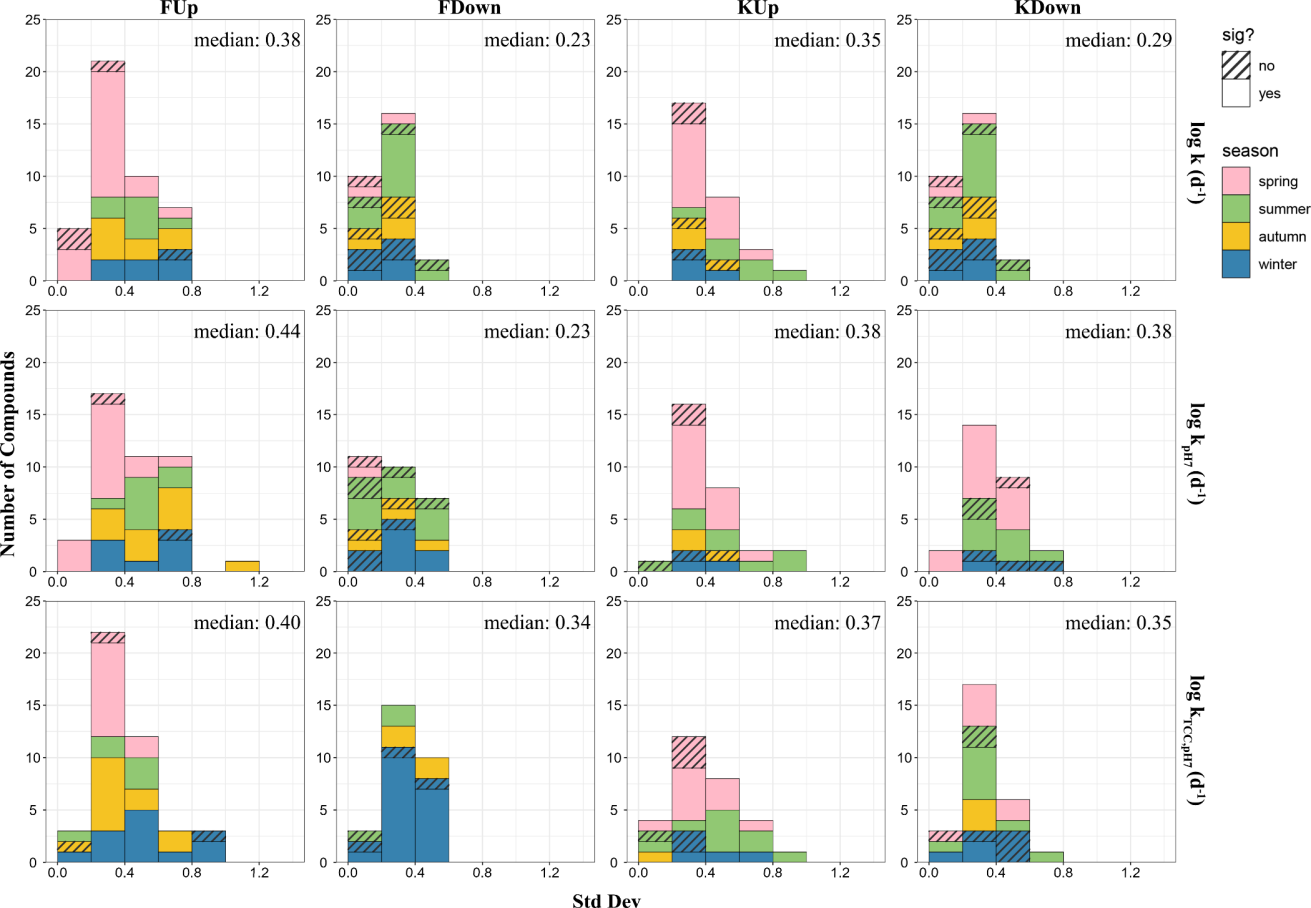
Frequency distribution histogram showing the standard deviation (Std Dev) of log *k* (d^–1^, upper row), log *k*_pH7_ (d^–1^, middle row), and log *k*_TCC,pH7_ (d^–1^, lower row)* across the 4 seasons, provided for each of the four experiments. The frequency histogram shows the number of compounds in each 0.2 log unit bracket of standard deviation. The color of the bar indicates the season with the maximum *k* (fastest biodegradation) for the compounds that fall in that bracket. A solid colored bar indicates a significant difference (sig) between the 4 seasons (ANOVA test, *p* < 0.05), while a striped pattern indicates that the difference was not significant. The median value of the standard deviation of log *k* for all compounds in the experiment is also shown. *k: biodegradation rate constant; *k*_pH7_: biodegradation rate constant at pH 7; *k*_TCC,pH7_: biodegradation rate constant at reference condition (pH = 7, TCC = 10^9^ cells per test flask (equivalent to 2.9 × 10^9^ cells L^–1^ test slurry)).

The observed *k* varied from 0.004 to 14.5 d^–1^ (except for decylamine, which had much higher *k* values up to 15.6, 18.8, and 26.7 d^–1^ in 3 seasonal experiments at 3 different locations). Maximal variation in *k* of up to 3 orders of magnitude between seasons and up to 2 orders of magnitude between rivers upstream and downstream was observed for individual chemicals. To describe the extent of the seasonality of *k*, the standard deviation of the logarithm of *k* (log *k*) was calculated for each compound that biodegraded in all 4 seasons (43 compounds in FUp, 28 in FDown, 29 in KUp, and 27 in KDown). It varied from 0.05 to 0.96 log units, with median values of 0.23–0.38 across the four sites (Figure 1). Of these compounds, 91% and 61% showed significant differences (ANOVA, *p* < 0.05) between seasons in FUp and FDown respectively, while 83% and 78% showed significant differences between seasons in KUp and KDown (Figure 1, Figure S8). The significant seasonality of *k* observed for most compounds at 4 different locations and the high median standard deviations (0.23–0.38 log units amounts to a factor of 1.7–2.4) indicates that the seasonal variability is large enough to markedly impact the chemical fate in some cases.

Most of the studied compounds biodegraded fastest in spring or summer (Figure 1) when the stream temperatures were highest (17 and 19 °C). However, 4% to 25% of the compounds had significantly faster biodegradation in autumn or winter (11 and 4 °C), suggesting that the seasonality of *k* for some compounds might be more strongly influenced by other environmental or microbial parameters than temperature. In both of the rivers, the median value of the standard deviation of log *k* across the 4 seasons was higher upstream of the WWTP than downstream (0.38 vs 0.23 in F, 0.35 vs 0.29 in K, Figure 1), indicating a higher seasonality of k upstream of WWTP outfalls.

### Seasonal Variation in pH-Dependent Speciation Corrected *k*

There was a pronounced seasonal variation in pH at the sampling locations ranging up to 0.9 pH units (Figure S2). Out of 96 biodegraded compounds, 91 are ionizable chemicals, and the estimated neutral fraction of 54% to 61% of them varied significantly over the 4 seasons at the individual locations (the relative standard deviation of the neutral fraction was >20%). Given the strong evidence indicating that changes in the neutral fraction will change the bioavailability of a substance for biodegradation, the observed *k* was corrected to a reference pH of 7 by multiplying k by the ratio of the neutral fraction at pH 7 to that at the mean pH in the test treatment to obtain *k*_pH7_ (SI S5):
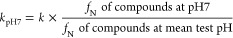


By conversion of *k* to a reference pH, the influence of changes in the neutral fraction on *k* can be largely eliminated, and *k*_pH7_ can be used to explore other causes for seasonal variability of biodegradation rates. After pH correction, the median standard deviation of log *k*_pH7_ increased slightly to 0.44 in FUp and 0.38 in both KUp and KDown, while it remained unchanged in FDown. 95% and 64% of the studied compounds still showed significant differences (ANOVA, *p* < 0.05) between 4 seasons in FUp and FDown, respectively, and 83% and 78% of compounds showed significant differences between seasons in KUp and KDown, respectively (Figure 1, Figure S8). The results demonstrate that the strong seasonal variability in the biodegradation rate constants was not a consequence of pH-dependent changes in the bioavailability of the ionizing compounds.

### Association Between Total Cell Count (TCC) and *k*_pH7_

A seasonal pattern was observed for field TCC at all 4 sampling locations (SI S2). The standard deviations of log TCC were 0.15 and 0.21 in FUp and FDown, and 0.16 and 0.05 in KUp and KDown across 4 seasons, respectively. The field TCC was reproducible between replicate measurements (relative standard deviation <15%) and maintained a similar level for all seasons at the same location (Table S4). Field TCC was 1.5 to 4 times higher in KDown than in FDown, which corresponds to the lower dilution of wastewater effluent in KDown.

To explore the influence of TCC on the seasonal variability of biodegradation rates, we assumed that *k* and field TCC were linearly related and converted *k*_pH7_ to a TCC reference state, which was based on the global average of cell densities in surface waters (∼10^6^ cells mL^–1^^16^), which corresponds to approximately 10^9^ cells per incubation flask. This conversion was done by multiplying *k*_pH7_ by the quotient of 10^9^ cells flask^–1^ and field TCC to obtain *k*_TCC, pH7_ (for more details see SI S5).
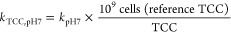


**Figure 2:**
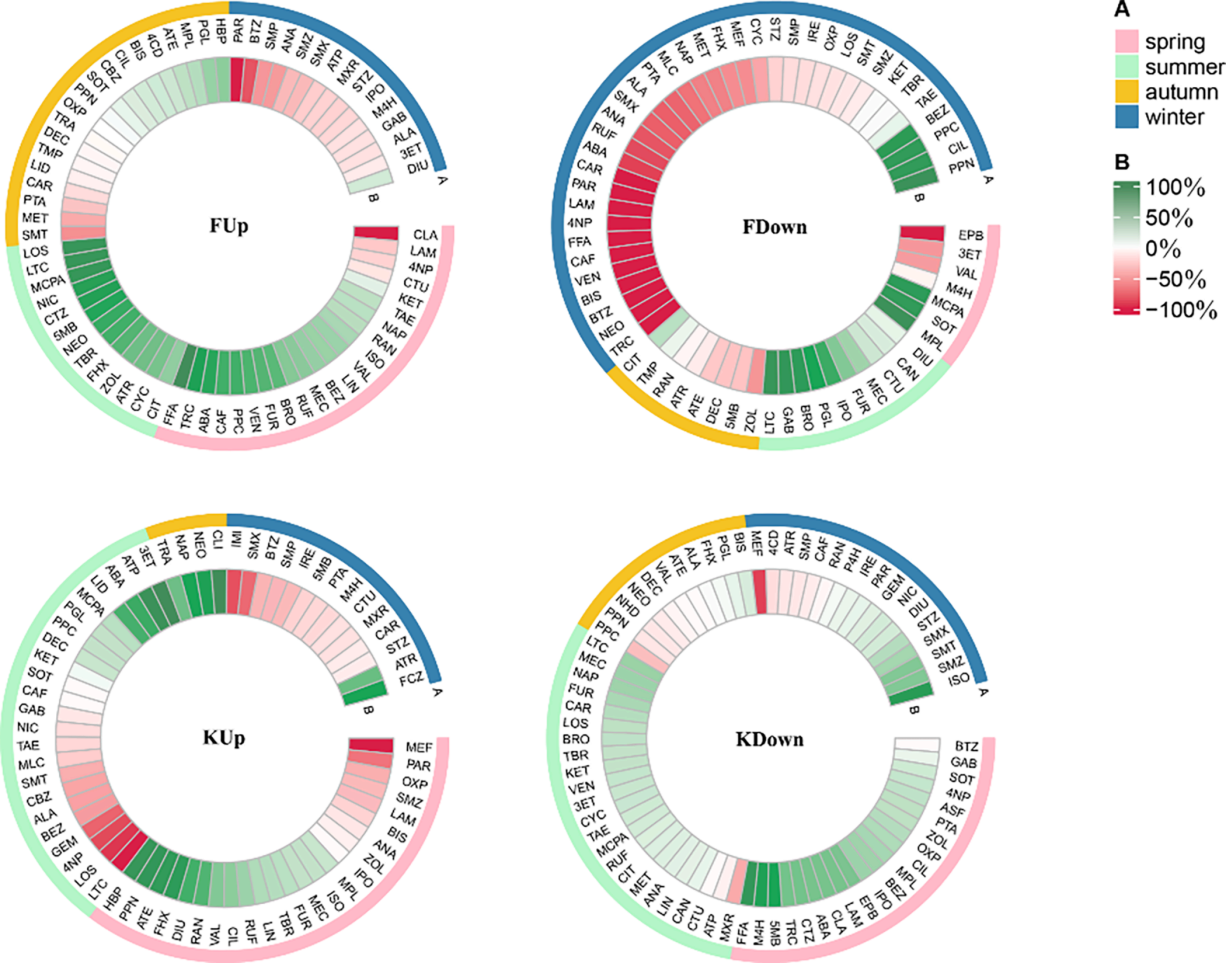
Circle heatmaps indicate for each chemical the season with maximum k_TCC,pH7_ (A), the reduction efficiency (RE, %) of the TCC normalization (B). , green: StdDev was reduced, red: StdDev was increased. RE was calculated for all compounds that biodegraded in at least two seasons. For elaboration of the compound name abbreviations, see Table S1.

If TCC were to explain a major portion of the variability in *k*, then *k*_TCC,pH7_ should be less variable than *k*_pH7_. The reduction efficiency (RE, %) of the TCC normalization was used to show the portion of the variability in *k*_pH7_ that could be explained by TCC:
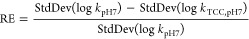


After correcting for both the neutral fraction and TCC, the percentage of compounds showing significant differences in *k*_TCC,pH7_ between the 4 seasons (ANOVA, *p* < 0.05) decreased slightly to 93% in FUp, 79% in KUp, and 74% in KDown, respectively, while in FDown the percentage increased to 86% (Figure 1, Figure S8). The median values of the standard deviation of normalized log *k* increased by 0.11 in FDown and decreased very little (0.01 to 0.04) in the other three locations (compare log *k*_TCC,pH7_ and log *k*_pH7_ in Figure 1). Clearly, the seasonal variation in TCC does not explain the observed seasonality of the biodegradation rate for the data set as a whole. However, for individual compounds that biodegraded in at least two seasons, the standard deviation of log *k*_pH7_ decreased by up to 95% following TCC normalization (Figure 2). The standard deviation of log *k*_pH7_ was reduced for 76% and 61%, of the compounds in KDown and FUp, respectively, while in KUp and FDown a reduction was only observed for 46% and 35% of the compounds. The results indicate that the seasonal variability of *k*_pH7_ showed an association with TCC for the majority of compounds in KDown and FUp, but not in KUp and FDown.

Figure 2 also provides insight into which seasons showed the strongest relationship between *k*_pH7_ and TCC. A reduction in the standard deviation of log *k*_pH7_ following TCC normalization was most common for chemicals for which *k*_TCC,pH7_ was the highest in spring or summer. For 81% to 93% of the compounds that had maximum *k*_TCC,pH7_ in winter, TCC normalization led to an increase in seasonal variability in FUp, FDown, and KUp, while in KDown, only 3 out of 17 compounds that had maximum *k*_pH7_ in winter showed a decrease >15%. Despite this strong association between maximum rate constants in winter and an increase in seasonality as a result of TCC normalization (i.e., a lack of correlation between *k*_pH7_ and TCC), there were hardly any individual chemicals for which this behavior was consistently observed across sites.

TCC normalization can explain variability in *k* if either the transformation reaction in question can be catalyzed by many different microorganisms (e.g., by a generally available, abundant enzyme)^42,43^ or if the abundance of more specialized enzymes/microorganisms responsible for the biodegradation is correlated with TCC. Since the latter seems unlikely given that pronounced seasonality in the diversity and composition of bacterial communities has been demonstrated,^8,11,26^ the absence of a correlation between seasonality of *k*_pH_ and TCC might therefore point toward compounds biodegraded by specific enzymes/microorganisms. Some compounds that did not show such a correlation in our study (VAL, CAF, and CYC in FDown and VAL, CAF in KDown) have been observed to be subject to biodegradation mediated by bacteria released from WWTPs.^12^ In addition, in a simulation test of hydrocarbon biodegradation in surface water at winter temperatures, no biodegradation was seen when the surface water was sampled in summer, while biodegradation was seen when the surface water was sampled in winter.^5^ This is consistent with the indications of specific biodegradation in the winter for some chemicals in our study (Figure 2).

### Temperature-Dependence of Biodegradation Kinetics under Environmentally Relevant Conditions

The Arrhenius equation was used to assess the temperature dependence of *k*, *k*_pH7_, and *k*_TCC,pH7_ at each station. About 10% to 40% of the cases had a strong negative correlation between 1/*T* (K) and log *k* or log *k*_pH7_, and 2% to 45% of the cases had a strong negative correlation between 1/*T* (K) and log *k*_TCC,pH7_ in FUp, KUp, and KDown (*R*^2^ > 0.7, Table S9). For those cases showing a strong negative correlation in FUp and KUp, 44% and 64% had an apparent activation energy (*E*_a_) whose 95% confidence interval intersected with the recommended ECHA value (65.4 kJ/mol), whereas this was observed for only 11% of the cases in KDown. In FDown, only 7% and 11% of the cases had a significant Arrhenius-type relationship for both *k* and *k*_pH7_, respectively, and only one case had a significant Arrhenius-type relationship for *k*_TCC,pH7_. Hence, most of the studied compounds did not show an Arrhenius-type behavior. This may be a consequence of the seasonal adaptation of bacteria^11,25,26^ having an impact on the biodegradation capacity of an ecosystem.^5^ Although there is evidence showing that the biodegradation half-lives of some chemicals determined at different test temperatures were consistent with the Arrhenius equation,^22^ it is important to realize that using the classical Arrhenius relationship to temperature-correct biodegradation rates does not necessarily improve the estimate of persistence of a given compound in the natural environment.

### Seasonal Pattern of *k*_TCC,pH7_ of Individual Compounds

**Figure 3:**
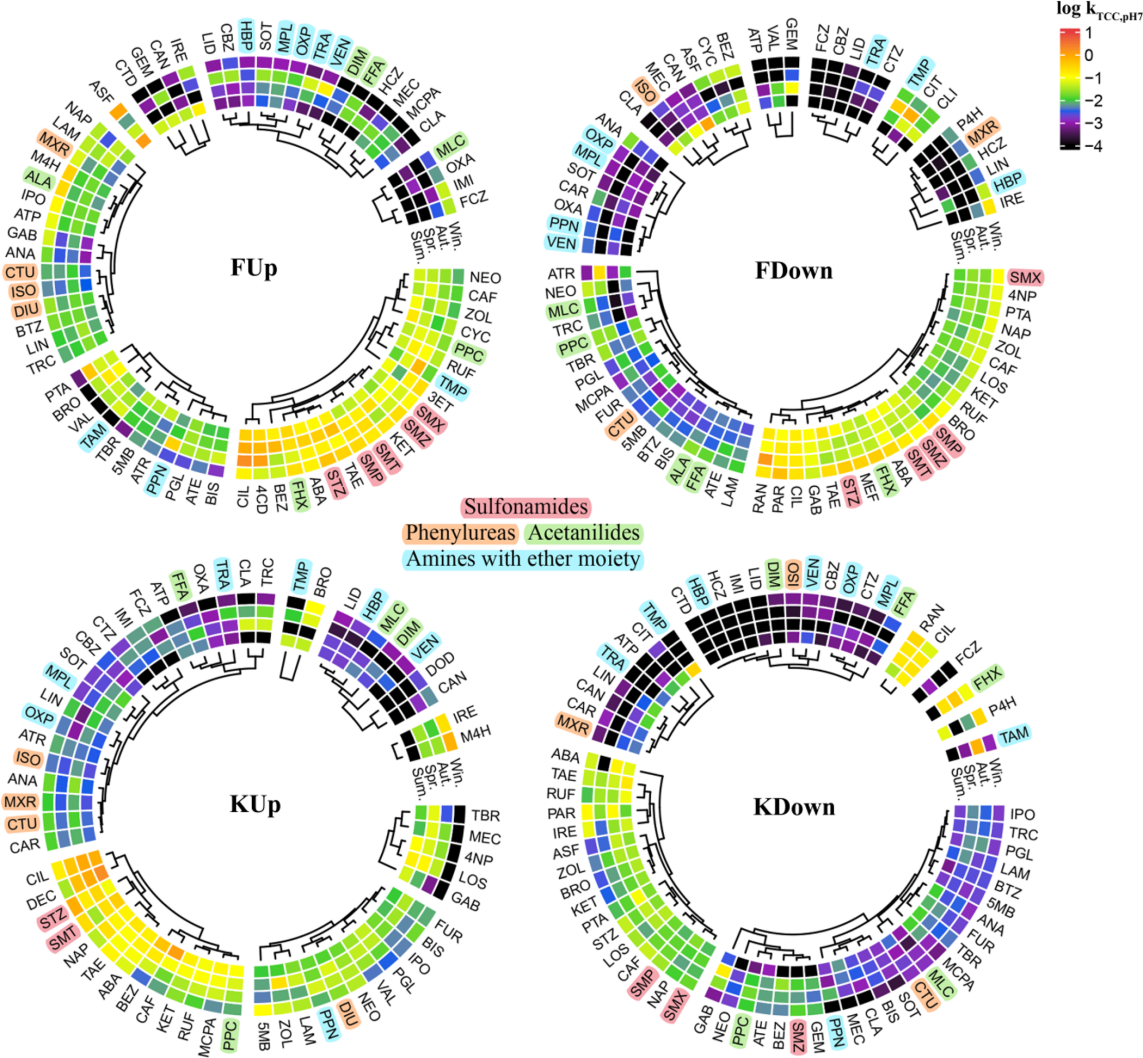
Clustered heatmaps showing log *k*_TCC,pH7_ (d^–1^) of the studied compounds. The clusters indicate compound groups showing a similar seasonality of *k*_TCC,pH7_ and a similar level of *k*_TCC,pH7_. All of the estimated *k* values were used for comparison regardless of whether or not they were significantly different from 0 to obtain more information. Only compounds with *k*_TCC,pH7_ available for all 4 seasons were used for clustering. When the log *k*_TCC,pH7_ was < −4 or a negative *k* was not significantly different from 0 (p-value >0.05), log *k*_TCC,pH7_ was set to −4. From the outer ring to the inner ring, the results are shown in the order of winter (Win.), autumn (Aut.), spring (Spr.), and summer (Sum.). Chemicals sharing specific functional groups were assigned a color, and this color was used to highlight the chemical name abbreviations. For elaboration of the compound name abbreviations, see Table S1.

To better understand the seasonality of biodegradation kinetics at an individual chemical level, the magnitude of *k*, *k*_pH7_, and *k*_TCC,pH7_ across seasons was assessed by hierarchical clustering (Figure 3, Figure S7). We found chemical-class-specific patterns of seasonality that were consistent for some chemical groups across locations. In this discussion, we focus on *k*_TCC,pH7_ because the normalization to the neutral fraction and TCC is believed to have eliminated much of the influence of speciation and active biomass on the rate constants, so that the influence of chemical structure on degradability should be more visible. We classify the magnitude of *k*_TCC,pH7_ as slow (<0.01), moderate (0.01 to 0.1), and fast (>0.1). The magnitude of *k*_TCC,pH7_ of compounds in each cluster relative to that in other clusters was generally consistent across seasons.

Figure 3 shows the results for *k*_TCC,pH7_ when only chemicals with data for all four seasons were included, while Figure S7 shows the results for all three metrics (*k*, *k*_pH7_, and *k*_TCC,pH7_) when chemicals with data for at least one season were included. Some compounds sharing specific functional groups cluster closely at each of the four locations, e.g., sulfonamides (SMX, SMT, SMZ, SMP, and STZ) and amines that also contain an ether moiety (AMI, FXT, HBP, MPL, OXP, PPN, TAM, TMP, TRA, and VEN). For the two sites upstream of the WWTPs, most of the phenylureas (CTU, DIU, ISO, and MXR) also cluster together. Some compounds sharing the same functional group do not cluster closely at all 4 locations, e.g., the acetanilides (ALA, DIM, MLC, PPC, FFA, and FHX). Clustering results for chemicals sharing other functional groups are shown in Figure S7.

It has been suggested that the biodegradation rates of organic contaminants sharing similar functional groups might covary because they are transformed by similar enzymes.^16,35^ Our results show similarities in both magnitude and seasonality of *k*_TCC,pH7_ within some groups of chemicals sharing specific functional groups but not for others. One explanation may be that some chemicals hold multiple functional groups, making classification difficult. A better approach may be to classify chemicals according to rate-limiting structural features or observed biotransformation pathways.^37^ For example, the environmental contaminant biotransformation pathway resource (enviPath)^44,45^ that relies on a curated database of biotransformation rules to predict the initial biodegradation pathway could be used to more confidentially assign the chemicals to groups sharing biotransformation reactions (and hence potentially degraded by the same enzyme groups). More work is warranted to understand the relationship between the molecular structure and changes in biodegradation rates under different environmental conditions.

### Comparison of Biodegradation Kinetics Up- and Downstream of WWTPs

Using individual *k*, *k*_pH7_, and *k*_TCC,pH7_ values determined for the three incubation replicates, we did paired comparisons of *k*_TCC,pH7_ upstream and downstream of the two WWTPs (F and K) to assess the impact of WWTP effluent on the biodegradation capacity in rivers. In addition to the lower seasonality of biodegradation downstream of WWTPs (Figure 1), we also found that more compounds showed slower biodegradation downstream (Figure 3, Figure S7). In 38% of the cases at both the F and K sites, *k* and *k*_pH7_ were significantly different between up- and downstream (paired *t* test for each season using the *k* for each replicate, *p* value <0.05), and in about 70% of these cases at F and 60% at K, the rate constants were lower downstream than upstream. *k*_TCC,pH7_ was significantly different between up- and downstream in 43% and 54% of the cases at the F and K sites, respectively (paired *t* test, *p* value <0.05), while in 80% and 94% of these cases, respectively, the compound degraded slower downstream than upstream. Some consistency in this behavior across compound groups was observed. For instance, phenylureas (CTU, DIU, ISO, MXR) always displayed faster biodegradation upstream of WWTPs than downstream (Figure 3, Figure S7). The TCC was always higher downstream of the WWTPs than upstream (∼1.5 times at F and ∼4 times higher at K (Table S4)), which is in agreement with observations at two WWTPs in Switzerland.^30^ The results indicate that the release of treated wastewater did not increase but rather decreased the biodegradation capacity of the downstream bacterial communities.

We looked more closely at the influence of concentration in river water on k. Out of 96 studied compounds, 20, 9, 5, and 2 chemicals were detected at a concentration above the spiking concentration of 1 μg L^–1^ in the river water from FDown, KDown, KUp, and FUp, respectively (section S8 of SI). In contrast to most of the compounds, *k*_TCC,pH7_ of some of the compounds present at high concentrations (>1 μg L^–1^) was greater downstream of WWTPs than upstream. For some of these compounds, there was an association between their concentrations in water and *k*_TCC,pH_ (Figure S9), which is in agreement with Desiante et al.^30^ For instance, candesartan was only detected downstream of WWTPs, it had the maximum *k*_TCC,pH_ in the summer at both sites when its concentration was much higher than in the other 3 seasons, and it was usually not biodegraded (*k* was not significantly different from 0) in the upstream incubations. A positive relationship between the chemical’s concentration and its *k*_TCC,pH_ was also found for some other studied compounds that had high concentrations downstream (CAF, CAN, MET, GEM, and PAR), as discussed in section S8 of the SI. The results indicate that, for a few compounds, higher concentrations could result in faster biodegradation downstream of WWTPs.

### Implications and Perspective

We assessed the seasonality of the biodegradation rates of 96 compounds in 4 river sections and demonstrated a significant seasonal variation in *k* for 61%–91% of the compounds. For individual substances, *k* varied by up to 3 orders of magnitude between seasons, and this could not be explained by seasonal variation in pH-dependent bioavailability or cell density. Clearly, taking seasonality into account could improve our understanding of chemical exposure. Furthermore, we found that the biodegradation behavior of most studied compounds did not follow an Arrhenius relationship with temperature. Consequently, other factors must be the dominant source of seasonal variability in the biodegradation rates of chemicals in rivers. Bacterial community structure and functions are factors that have been shown to have seasonal variation^11,25,26^ and an association with temporal variability of *k*.^8^ The abundance of some bacterial strains varies between seasons in ways that are not necessarily correlated with temperature.^26^ In our study, we observed a specific biodegradation in the winter, which may hint at a biodegradation associated with the winter-adapted bacterial community. To address this hypothesis, we need more research on the association between microbial communities and the rate of chemical biodegradation.

Using the Arrhenius equation to extrapolate biodegradation rates from 12 °C (standard) to another temperature was recommended by ECHA for predicting the persistence of chemicals in the environment.^24^ However, in our work, the biodegradation of multiple compounds deviated from the classical Arrhenius-type behavior at all 4 study sites. The results are consistent with previous findings^5,8^ and suggest that the variation in the microbial community,^11^ rather than temperature, might dominate the variability of biodegradation rate in the natural environment. Therefore, using the Arrhenius model to predict biodegradation at different temperatures without accounting for differences in the microbial community may result in misestimation of chemical persistence and potentially poor regulatory decisions.

Interestingly, we found similarities in the magnitude and seasonality of *k*_TCC,pH_ for some groups of compounds that share a specific functional group. This suggests that chemical group-specific benchmarking techniques^3,46^ may provide a path to better describe the variability of biodegradation rates in space and time. Our understanding could be improved further by identifying the main enzymatic transformation reactions for multifunctional compounds and developing better bioinformatics pipelines to connect observed reactions to data from molecular microbiology (e.g., functionally annotated sequencing information).

The current study also shows the impact of wastewater discharge on the biodegradation of chemicals in water-sediment systems. In addition to reduced seasonality of biodegradation downstream, our results show that for many compounds, *k* was not significantly different between up- and downstream of WWTPs. About one-third of the studied compounds biodegraded more slowly downstream than upstream, while a few other compounds showed much faster biodegradation downstream when their concentration in water was high (caffeine, candesartan, gemfibrozil, metformin, and paracetamol). Knowledge of the effects of anthropogenic pollution on the biodegradation of individual compounds is important for assessing the robustness and health of surface water ecosystems.

Finally, before seasonality can become a common component of chemical exposure assessment, we need a better understanding of the spatial variability in biodegradation rates and how the rate constants measured in incubation tests relate to the rate constants for primary biodegradation in real aquatic systems.

## Supporting Information Available

Biodegradation kinetic parameters of quantified test chemicals (XLSX)
Table S1: list of test compounds; Table S2: list of internal standards; Table S3: targeted compounds that were problematic and excluded from further analysis; Table S4: total cell count; Table S5: mean of all pH values measured in the 3 replicate test incubations in each experiment; Table S6: number of chemicals degraded, persistent or for which data were not available for each of the experiments; Table S7: description of the categories classified based on the result of the Chow test; Table S8: ratio between dissipation rate constants in test treatments and the sorption controls (SC) for chemicals showing a significant *k* and dissipation in SC in at least one experiment; Table S9: Arrhenius relationship between *chowclassifier* generated *k*, *k*_pH7_, and *k*_TCC,pH7_ and temperature (*T*, K); Figure S1: locations of the sampling sites; Figure S2: characteristics of the sampling site; Figure S3: field total cell counts measured in water and sediment in experimental vessels; Figure S4: RSD of the 96 targeted chemicals in the sorption control samples after drift correction, of the internal standards in all samples after drift correction, and of *k*; Figure S5: schema for the Chow classifier (*chowclassifier*); Figure S6: biodegradation kinetics of quantified test chemicals; Figure S7: clustered heatmaps showing log *k*, log *k*_pH7_, and log *k*_TCC,pH7_ of the studied compounds; Figure S8: heatmaps displaying the significance of the seasonality in *k*, *k*_pH7_, and *k*_TCC,pH7_, expressed as the log *p* value; Figure S9: correlation between k_TCC,pH7_, and concentration of the compound in the river (PDF)
