# Supplementary material for: Influence of Season on Biodegradation Rates in Rivers
Source: Environ Sci Technol. 2024 Mar 25;58(16):7144–53. doi: 10.1021/acs.est.3c10541 (PMC11044578; doi:10.1021/acs.est.3c10541)
Supplement: Supplementary file 2 — es3c10541_si_002.pdf [file es3c10541_si_002.pdf]

Influence of season on biodegradation rates in rivers

Run Tian,[1] Malte Posselt,[1] Luc T. Miaz, [1]Kathrin Fenner,[2,3] Michael S. McLachlan[1]*

[1]Department of Environmental Science (ACES), Stockholm University, 10691 Stockholm, Sweden

[2]Eawag, Swiss Federal Institute of Aquatic Science and Technology, 8600 Dübendorf, Switzerland

[3]University of Zürich, Department of Chemistry, 8057 Zürich, Switzerland

*Corresponding author: run.tian@aces.su.se

Table of Contents

S1. Additional information on materials and methods 5

S1.1 Test compounds 5

S1.2 Biodegradation experiments 10

S1.3 Characteristics of the sampling sites 11

S1.4 Problematic substances 13

S2. Total cell count analysis 14

S2.1 Enumeration of bacteria cells 14

S2.2 Total cell count (TCC) in the incubation system 14

S3. Quality control and quality assurance 16

S3.1 Environmental conditions 16

S3.2 Chemical analysis and experimental results 16

S3.3 Python algorithm (Chowclassifier) for recognizing the breakpoint in biphasic kinetics 18

S4. Abiotic dissipation 20

S5. Biodegradation kinetics of quantified test compounds 22

S6. Significant difference in the seasonality of the rate constants 42

S7. Arrhenius relationship between k, k_{pH7}, and $k_{TCC,pH7}$ and temperature (T, K). 43

S8. Seasonal biodegradation of high-concentration pollutants in rivers 46

List of Tables

Table S1. List of test compounds. 5
Table S2. List of internal standards 9
Table S3. Targeted compounds that were problematic and excluded from further analysis... 13
Table S4. Total cell count (*10E+9, cells) 15
Table S5. Mean of all pH values measured in the 3 replicate test incubations in each experiment 16
Table S6. Number of chemicals degraded, persistent or for which data were not available for each of the experiments 17
Table S7. Description of the categories classified based on the result of the Chow test 18
Table S8. Ratio between dissipation rate constants in test treatments and the sorption controls (SC) for chemicals showing a significant k and dissipation in SC in at least one experiment 21
Table S9. Arrhenius relationship between chowclassifier generated k, k_{pH7}, and $k_{TCC,pH7}$ and temperature (T, K) 43

List of Figures

Figure S1. Locations of the sampling sites 11

Figure S2. Characteristics of the sampling site 12

Figure S3. Field total cell counts measured in water and sediment in experimental vessels .. 15

Figure S4. RSD of the 96 targeted chemicals in the sorption control samples after drift correction, of the internal standards in all samples after drift correction, and of k 17

Figure S5. The schema for the chow classifier (chowclassifier) 19

Figure S6. Biodegradation kinetics of quantified test chemicals 39

Figure S7. Clustered heatmaps showing log k, log k_{pH7}, and log $k_{TCC,pH7}$ of the studied compounds. 41

Figure S8. Heatmaps displaying the significance of the seasonality in k, k_{pH7}, and $k_{TCC,pH7}$, expressed as the log p value 42

Figure S9. Correlation between $k_{TCC,pH7}$, and concentration of the compound in the river 49

S1. Additional information on materials and methods

S1.1 Test compounds

Test compounds addressed in this study were selected using the following criteria: (1) not volatile; (2) expected to be found in the wastewater that discharges into the river; (3) have a broad range of biodegradability; (4) sufficiently hydrophilic with low log D_{OW} (80% of the chemicals have a log $D_{OW} < 3$, at pH 7.4) and expected to display a low sorption potential to organic carbon. Based on those criteria, 129 test compounds were selected (Table S1), including pharmaceuticals, agrochemicals, cosmetics, food additives, and industrial chemicals. In addition, these compounds were selected to cover a range of initial biodegradation reactions according to existing literature (sulfonamides, thioethers, acetanilides, phenylureas, amides, amines, etc.).

The test chemicals were purchased from Sigma-Aldrich (Steinheim, Germany) and Toronto Research Chemicals Inc. (North York, Canada), or were gifts from Unilever. D- and ^{13}C-labeled chemicals were purchased from Toronto Research Chemicals Inc. and CDN Isotopes (Pointe-Claire, Quebec, Canada) for use as internal standards. A working solution containing all test compounds was prepared at a concentration of 1.75 μg mL^{-1} in Milli-Q water. An internal standard solution containing all isotope-labeled standards was prepared at a concentration of 1 μg mL^{-1} in methanol. All the solutions were stored in the dark at -20 °C until use. LC/MS-grade methanol was purchased from VWR (Stockholm, Sweden). LC/MS-grade formic acid was purchased from Sigma-Aldrich. Sodium azide (NaN_3) was purchased from Sigma-Aldrich. Milli-Q water was produced by using a Milli-Q Integral Water Purification System (Merck Millipore, Stockholm, Sweden).

Table S1. *List of test compounds. The abbreviations (Abbr.) of the compounds are used in Figures 2, 3 and Supporting Information Tables S8, S9, Supplemental Dataset S1, and Figures S6-S9.*

	**Compounds**	**Abbr.**	**CAS Number**	**log D_{OW}[2] (pH 7.4)**	**p*Ka*[3]**
1	1-Stearoyl-rac-glycerol	1ST	123-94-4	2.6	-
2	2,6-Di-tert-butyl-4-methylphenol	2DI	128-37-0	5.07	11.6*[a]
3	3-((2-Ethylhexyl)oxy)propane-1,2-diol	3ET	70445-33-9	2.4	13.64*[a]
4	3-Methyl-4-(2,6,6-trimethyl-2-cyclohexen-1-yl)-3-buten-2-one	3ME	127-51-5	4.22	-
5	4-(4-Nitrobenzyl)-pyridine	4NP	1083-48-3	2.6	5.51*[b]
6	4-Chloro-3,5-dimethylphenol	4CD	88-04-0	2.83	9.7[b]
7	5-Methylbenzotriazole	5MB	136-85-6	1.69	8.74*[b]
8	Abacavir	ABA	136470-78-5	1.32	5.8*[b]
9	Acesulfame	ASF	33665-90-6	-2.77	2.0[a]
10	Acetamiprid	ATP	135410-20-7	1.06	0.7[a]
11	Alachlor	ALA	15972-60-8	2.99	1.20*[a]
12	Amisulpride	AMI	71675-85-9	-0.43	9.37[b]
13	Anastrozole	ANA	120511-73-1	2.68	1.4[b]
14	Atazanavir	ATA	198904-31-3	4.61	4.42*[b]
15	Atenolol	ATE	29122-68-7	-1.85	9.58[b]
16	Atrazine	ATR	1912-24-9	2.66	4.2[b]

17	Azoxystrobin	AZO	131860-33-8	3.54	1.94*[b]
18	Benzotriazole	BTZ	95-14-7	1.5	8.37[b]
19	Benzyl alcohol	BEA	100-51-6	4.85	15.4[a]
20	Benzyl salicylate	BES	118-58-1	4.01	8.11*[b]
21	Bezafibrate	BEZ	41859-67-0	-0.11	3.83*[a]
22	Bisoprolol	BIS	66722-44-9	0.12	9.67*[b]
23	Bromoxynil	BRO	1689-84-5	3.77	3.86[a]
24	C12 Isethionate	CIS	7381-01-3	-	-
25	Caffeine	CAF	58-08-2	0.28	-1.2[b]
26	Candesartan	CAN	139481-59-7	0.04	2.45[b]
27	Carbamazepine	CBZ	298-46-4	2.28	13.9[a]
28	Carbendazim	CAR	10605-21-7	1.51	4.29[b]
29	Chlorothiazide	CTZ	58-94-6	-0.21	4.29[a]
30	Chlorthalidone	CTD	77-36-1	0.41	6.85[a]
31	Chlortoluron	CTU	15545-48-9	2.48	14.43*[a]
32	Cilastatin	CIL	82009-34-5	-1.9	9.14*[b]
33	Ciprofloxacin	CIP	85721-33-1	-2.23	8.77*[b]
34	Citalopram	CIT	59729-33-8	1.27	9.78[b]
35	Climbazole	CLI	38083-17-9	3.32	6.49[b]
36	Clofibric acid	CLA	882-09-7	-0.88	-
37	Cocoamidopropyl betaine	COC	61789-40-0	0.93	-
38	Cyclamate	CYC	100-88-9	-3.51	1.7[b]
39	Decylamine	DEC	2016-57-1	1.07	10.64[b]
40	Dibenzepin	DIB	4498-32-2	1.24	8.23[b]
41	Dicamba	DAB	1918-00-9	1.24	1.97[b]
42	Diclofenac	DIC	15307-86-5	1.37	1.97[a]
43	Diflufenican	DIF	83164-33-4	4.09	9.03*[b]
44	Dimethenamid	DIM	87674-68-8	2.45	1.16[b]
45	Dioctyl Sulfocinate Sodium Salt	DIO	10041-19-7	1.01	-0.75*[a]
46	Diuron	DIU	330-54-1	0.13	-
47	Dodecyl sulfate sodium salt	DSS	151-21-3	1.6	-1.5[a]
48	Dodecylamine	DOD	124-22-1	1.86	10.63*[b]
49	Dodecyltrimethylammonium chloride	DOC	112-00-5	-	-
50	Ethofumesate	EFS	26225-79-6	2.14	-
51	Ethylene glycol butyl ether	EGB	111-76-2	0.77	14.42*[a]
52	Ethylhexyl methoxycinnamate	EMC	5466-77-3	5.28	-4.8*[b]
53	Ethylparaben	EPB	120-47-8	2.48	8.34[a]
54	Fenhexamid	FHX	126833-17-8	4.17	7.3[a]
55	Fenofibrate	FFE	49562-28-9	5.01	-4.9*[b]
56	Fipronil	FIP	120068-37-3	3.71	-5.86[b]
57	Flecainide	FLE	54143-55-4	1.01	9.3[b]
58	Fluconazole	FCZ	86386-73-4	0.7	1.76[b]
59	Fludioxonil	FDO	131341-86-1	2.57	14.10[a]
60	Flufenacet	FFA	142459-58-3	3.01	0.31*[a]
61	Fluoxetine	FXT	54910-89-3	1.75	9.8*[b]

62	Furosemide	FUR	54-31-9	-0.78	3.65[a]
63	Gabapentin	GAB	60142-96-3	-1.4	3.7[a]
64	Galaxolide	GAL	1222-05-5	5.93	-
65	Gemfibrozil	GEM	25812-30-0	1.58	4.5[a]
66	Hexylene glycol	HGL	107-41-5	0.23	15.10[a]
67	Homosalate	HOM	118-56-9	5.23	9.72*[a]
68	Hydrochlorothiazide	HCZ	58-93-5	-0.01	7.9[a]
69	Hydroxy bupropion	HBP	357399-43-0	1.93	-
70	Imidacloprid	IMI	138261-41-3	-0.29	1.56[b]
71	Iodopropynyl butylcarbamate	IBC	55406-53-6	3.2	14.4*[a]
72	Iprovalicarb	IPO	140923-17-7	3.29	11.41*[a]
73	Irbesartan	IRE	138402-11-6	1.24	5.85*[a]
74	Isoproturon	ISO	34123-59-6	2.45	15.06*[a]
75	Ketoprofen	KET	22071-15-4	0.06	3.98[a]
76	Lamotrigine	LAM	84057-84-1	1.68	5.7[b]
77	Levamisole	LAS	14769-73-4	0.25	6.98*[b]
78	Levetiracetam	LTC	102767-28-2	-0.74	-1.6*[b]
79	Lidocaine	LID	137-58-6	1.26	7.95[b]
80	Linezolid	LIN	165800-03-3	0.82	1.8[b]
81	Losartan	LOS	114798-26-4	1.29	5.5[a]
82	Loxynil	LOX	1689-83-4	1.27	3.96[a]
83	MCPA	MCPA	94-74-6	-1.09	3.13[a]
84	Mecoprop	MEC	7085-19-0	-0.65	3.21[a]
85	Mefenamic acid	MEF	61-68-7	2.04	4.2[a]
86	Metformin	MET	657-24-9	-3.36	12.4[b]
87	Methotrexate	MEX	59-05-2	-5.22	4.7[a]
88	Methyl 4-hydroxybenzoate	M4H	99-76-3	2.09	8.5[a]
89	Metolachlor	MLC	51218-45-2	3.22	1.45[a]
90	Metoprolol	MPL	51384-51-1	-0.25	9.56[b]
91	Metoxuron	MXR	19937-59-8	1.87	13.83[a]
92	N-(3-dimethylaminopropyl)-octadecanamide	NDO	7651-02-7	6.12	16.29*[a]
93	N,N-bis(2-hydroxyethyl)dodecanamide	NHD	120-40-1	3.95	14.13[a]
94	N,N-Bis(2-hydroxyethyl)tetradecanamide	NHT	7545-23-5	4.87	-
95	Naphthalene-2-carbonitrile	N2C	613-46-7	3.22	-
96	Naproxen	NAP	22204-53-1	0.45	4.15[a]
97	Neostigmine	NEO	59-99-4	-2.77	-
98	Nicotinamide	NIC	98-92-0	-0.37	3.35[b]
99	Novobiocin	NOV	303-81-1	-0.02	4.3[a]
100	Oxazepam	OXA	604-75-1	1.5	1.55[b]
101	Oxprenolol	OXP	6452-71-7	0.19	9.57[b]
102	Paracetamol	PAR	103-90-2	0.4	9.38[b]
103	Pargyline	PGL	555-57-7	2.31	8.05*[b]
104	Picoxystrobin	PIC	117428-22-5	3.84	-1.09*[a]
105	Propachlor	PPC	1918-16-7	2.28	0.30*[a]

106	Propranolol	PPN	525-66-6	1.15	9.53[b]
107	Propyl 4-hydroxybenzoate	P4H	94-13-3	2.81	8.5[a]
108	p-Toluenesulfonic acid	PTA	104-15-4	-3.36	-1.34[a]
109	Ranitidine	RAN	66357-35-5	-0.63	7.8*[b]
110	Rufinamide	RUF	106308-44-5	0.42	12.64[a]
111	Sodium decyl sulfate	SDS	142-87-0	-	-
112	Sorbitan monolaurate	SML	1338-39-2	4.02	-
113	Sotalol	SOT	3930-20-9	-1.63	9.76[a]
114	Sulfadimethoxine	SMX	122-11-2	-0.49	6.91*[a]
115	Sulfamethazine	SMT	57-68-1	0.79	7.59[a]
116	Sulfamethoxazole	SMZ	723-46-6	-0.56	6.16*[a]
117	Sulfamethoxypyridazine	SMP	80-35-3	-0.29	6.84*[a]
118	Sulfathiazole	STZ	72-14-0	0.03	7.2[a]
119	Tamsulosin	TAM	106133-20-4	0.77	9.28*[b]
120	Tartrazine	TAR	1934-21-0	-10.17	-
121	Terbutryn	TBR	886-50-0	1.38	4.30[b]
122	Tetradecylamine	TAL	2016-42-4	3.05	10.62[a]
123	Tramadol	TRA	27203-92-5	0.52	9.41[b]
124	Triethyl citrate	TRC	77-93-0	1.09	11.82[a]
125	Trimethoprim	TMP	738-70-5	-1.15	7.12[b]
126	Trinexapac-ethyl	TAE	95266-40-3	-1.76	4.7[a]
127	Valsartan	VAL	137862-53-4	-0.89	3.6[a]
128	Venlafaxine	VEN	93413-69-5	1.43	9.5[b]
129	Zolpidem	ZOL	82626-48-0	3.06	5.65[b]

[1] It was difficult to distinguish 5-methylbenzotriazole from its isomers. Since the feature with retention time and mass spectrum in all 4 seasonal experiments was similar to that in calibration standards, the feature was assumed to be 5-methylbenzotriazole for subsequent comparisons.

[2] log *Dow* were obtained from ChemSpider.

[3] p*K*a was obtained from PubChem, and the values with an asterisk were predicted values obtained from DrugBank and ChemBK. [a] acid pKa, [b] base pKa.

Table S2. *List of internal standards.*

	**Compounds**
1	2-hydroxy-Ibuprofen-d6
2	acesulfame-d4
3	acetaminophen-d4
4	atenolol-d7
5	atorvastatin-d5
6	bezafibrate-d4
7	caffeine-d9
8	climbazole-d4
9	carbamazepine-d8
10	clofibric acid-d4
11	conitine-(methyl-d3)
12	fluconazole-d4
13	gabapentin-d6
14	gemfibrozil-d6
15	glimepiride-d5
16	irbesartan-d6
17	ketoprofen-^{13}C, d3
18	MCPA-d6
19	mecoprop-d3
20	metformin-d6
21	metoprolol acid-d5
22	metoprolol-d7
23	naproxen-d3
24	oxazepam-d5
25	pravastatin-d3
26	propranolol-d7
27	sulfamethoxazole-d4
28	tramadol-d6
29	triethyl-d15-phosphate
30	valsartan-d3
31	venlafaxine-d6
32	zolpidem-d6

S1.2 Biodegradation experiments

Sampling was carried out in 2 Swedish rivers (Vitsån, Knivstaån) up- and downstream of wastewater treatment plants (WWTP): FUp: upstream of Fors WWTP, FDown: downstream (~700 m) of Fors WWTP, KUp: upstream of Knivsta WWTP, KDown: downstream (~500 m) of Knivsta WWTP during 4 seasons (winter: 2022.03.17, spring: 2022.06.08, summer: 2022.08.01, 2022.10.04). The upstream river sections received no wastewater input. The downstream sampling sites were far enough from WWTP outfalls to ensure complete mixing of effluent with river water and similar water temperature to upstream. Water samples were collected 20 cm below the surface and sediment was collected by taking the top 3-5 cm layer with a sediment core sampler. Multiple sediment samples were transferred into two separate 2-liter amber glass bottles and mixed before sieving. All the samples were transported in a cooled and insulated container from the sampling sites to the lab. The incubations were started within 24 hours of sampling. The setup of the biodegradation simulation test followed the protocol of the modified OECD 309 test developed by our group to improve the test's environmental relevance.[1] All experiments were carried out in the dark at river water temperature. Since all sampling upstream and downstream of the two rivers was conducted on the same day, the variation in water temperature between river sites was within the expected range of temperature fluctuation during the day, and therefore all experiments for a given season were conducted in one temperature-controlled room simultaneously (winter: 4°C; spring: 17°C; summer: 19°C; autumn: 11°C). Sediment was sieved to 2 mm and homogenized. 17.5 g were added to a 500 mL Erlenmeyer flask and 350 mL of river water was added to give a sediment concentration of 50 g wet solid L^{-1}. The incubators were sealed with parafilm to minimize changes in water pH caused by the outgassing of CO_2 from the oversaturated river water. An orbital shaker (KS 501 digital, IKA, about 150 rpm) was used to keep the sediment in suspension during the incubation. Test incubations (3 replicates), sorption controls (SC, sterilized sediment-water mixtures for distinguishing biodegradation from sorption, 2 replicates) and a hydrolysis control (sterilized river water for distinguishing biodegradation and sorption from hydrolysis) were spiked with an aqueous mixture of 129 compounds (including pharmaceuticals, industrial chemicals, agrochemicals, food additives, and cosmetics) to a concentration of 1 μg L^{-1} each. Prior to spiking, we compared the peak area of each chemical in the 1 μg L^{-1} samples produced by diluting the spiked aqueous solution (1.75 mg L^{-1} in MilliQ water) and the methanol stock solution (1.75 mg L^{-1} in methanol), respectively. The peak area was similar for the majority of the compounds between the samples made by the spiked aqueous solution and the methanol stock solution. Less than 15 compounds out of 129 compounds had an obvious difference (however, < 20%) in peak area. The incubators were shaken for 10 minutes before the start of the experiment to ensure complete mixing. Dissipation of the test compounds was monitored by analyzing subsamples taken from the water phase of each vessel after 0, 2, 5, 9, 18 hours, 1, 2, 4, 6, 8, and 10 days. The dissolved oxygen content (DO) was measured every day, and the pH and conductivity were measured at 0, 1, 2, 4, 7, and 10 days using hand-held probes (HQ portable meters, HACH). The sterilization was performed by adding sodium azide (NaN_3) at a final concentration of 0.1% to each abiotic incubation. All incubation vessels were covered with parafilm to maintain constant pH during the incubation.

Sub-samples of the sieved and homogenized sediment were collected before filling the incubation flasks for the determination of total organic carbon (TOC). They were immediately frozen and stored at -20°C until analysis. TOC analysis was done by Eurofins using the loss-on-ignition (LOI) method.

Sediment and water samples were also collected for the determination of the total cell count (TCC) using flow cytometry measurements (FCM). Samples were collected:

i) from the sieved and homogenized sediment and river water prior to filling the incubation

flasks (called “field cell density”);
ii) from a non-spiked test incubation, which was identical to the test incubation except that no chemical was spiked and it was sacrificed after 1 h of shaking (called “initial cell density”);
iii) from each incubation at the end of the experiment (called “final cell density”).
Sample preparation for cell density measurement was based on a study by Seller et al.[1] For the determinations of initial and final cell density, the suspended sediment in the incubation flask was allowed to settle, 5 mL of supernatant was transferred into a 20 mL amber glass vial containing 5 mL of 2% paraformaldehyde (PFA) solution buffered with 0.1% pyrophosphate. The remaining supernatant was removed, leaving the sediment in the flask. A total of 5 g wet sediment was transferred into a 20 mL amber glass vial containing 5 mL of buffered 2% PFA solution. The water and sediment from the field were preserved in the same way. All samples were stored at 4°C in the dark until analysis.

S1.3 Characteristics of the sampling sites

Knivstaån Vitsån

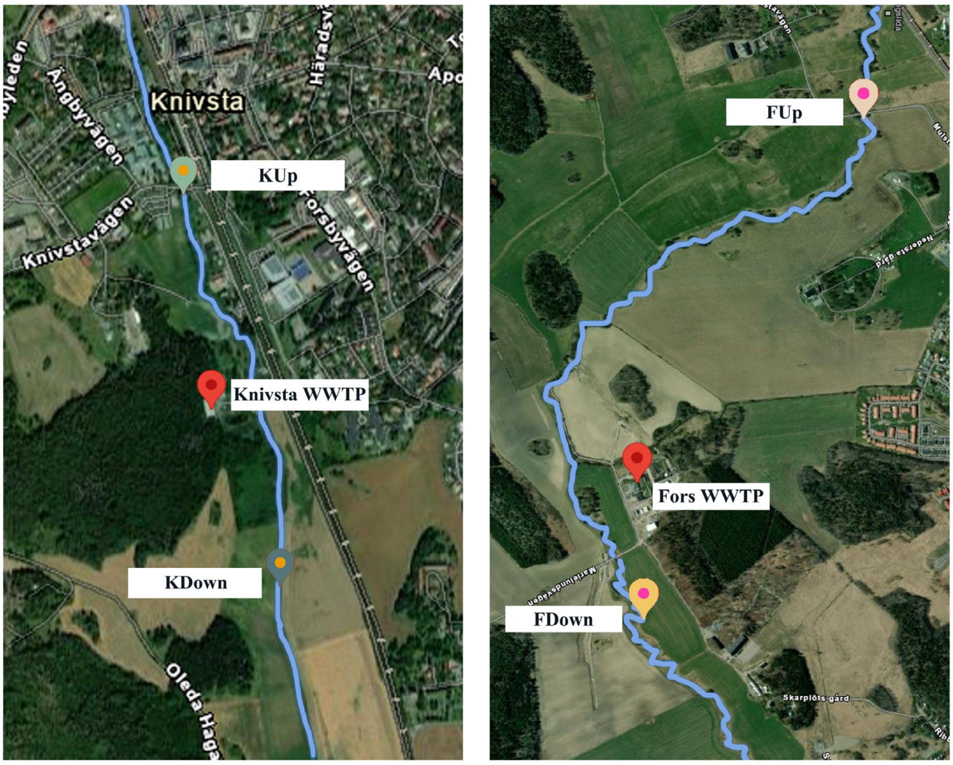

Figure S1. *Locations of the sampling sites. Maps are obtained from ArcGIS.*

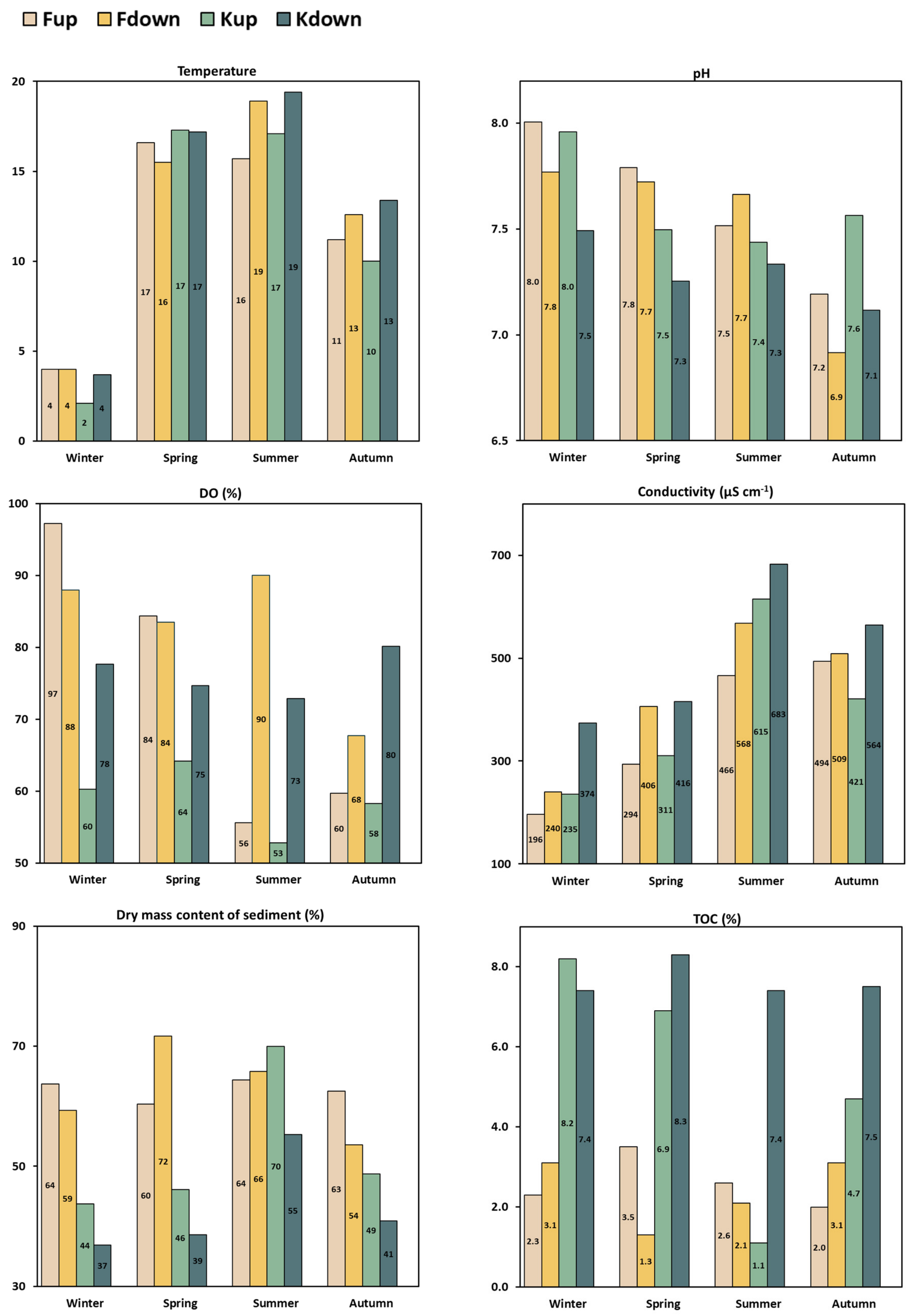

Figure S2. *Characteristics of the sampling sites (temperature, pH, DO, and conductivity of the water; dry mass content and TOC of the sediment). Temperature, pH, DO and conductivity were measured on-site.*

S1.4 Problematic substances

Table S3. *Targeted compounds that were problematic and excluded from further analysis.*

	**Compounds**
Targeted but not identified	2,6-Di-tert-butyl-4-methylphenol
	Benzyl alcohol
	Benzyl salicylate
	Ethylene glycol butyl ether
	Ethylhexyl methoxycinnamate
	Galaxolide
	Hexylene glycol
	Homosalate
	Sorbitan monolaurate
Identified but lower than LOD in treatment samples	1-Stearoyl-rac-glycerol
	3-Methyl-4-(2,6,6-trimethyl-2-cyclohexen-1-yl)-3-buten-2-one
	Cocoamidopropyl betaine
	Dioctyl Sulfocinate Sodium Salt
	Dodecyl sulfate sodium salt
	Iodopropynyl butylcarbamate
	N-(3-dimethylaminopropyl)-octadecanamide
	N,N-Bis(2-hydroxyethyl)tetradecanamide
	Naphthalene-2-carbonitrile
	Tartrazine
Detected in treatment samples but not quantified (lower than LOQ)	Atazanavir
	Azoxystrobin
	Diflufenican
	Ethofumesate
	Fenofibrate
	Fipronil
	Fludioxonil
	Loxynil
	Methotrexate
	Picoxystrobin
	Tetradecylamine
Significant dissipation in water abiotic control (W) thus not quantified in test treatments	Ciprofloxacin
	Dodecyltrimethylammonium chloride
	Sodium decyl sulfate

S2. Total cell count analysis

S2.1 Enumeration of bacteria cells

Enumeration of bacteria cells was performed by a BD Accuri C6 Flow Cytometer (BD, Belgium). The methods used for sampling and sample storage are described in the section on Biodegradation experiments (S1.2). Sample processing prior to analysis followed the methods developed by Seller et al.[1] Briefly, after shaking well, the water samples were transferred into Eppendorf tubes and diluted with Evian water which had been sterilized by filtration through a 0.2 μm polyethersulfone syringe filter. Bacterial cells in sediment samples were detached from particles by treatment with a 38 W ultrasonic needle probe for 80 seconds (4 cycles, 20 seconds each). Samples were cooled on ice during ultrasonic treatment. The supernatant solution containing detached cell suspension was transferred into Eppendorf tubes and diluted with sterilized Evian water.

The cells were stained using SYBR® Green I (Invitrogen AG, Basel, Switzerland) nucleic acid gel stain diluted in 10 mM Tris buffer (pH= 8) to 1:100 (v/v). Aliquots of 5 μL of the stain solution were added to 495 μL of the diluted sample. Stained samples were vortexed briefly and incubated at 37 °C for 15 min prior to analysis.

The instrumental threshold was set to 800 (FL1-H) and a volume of 50 μL was measured at high flow velocity (66 μL min^{-1}). Green and red fluorescence were measured at 533±30 nm (FL1-H channel) and >670 nm (FL3-H channel), respectively. Data were analyzed with the BD Accuri C6 Analysis Software 1.0.264.21. Total bacterial abundance was determined by enumeration of signals collected on the combined FL1/ FL3 density plot.

S2.2 Total cell count (TCC) in the incubation system

The TCC in the incubation flasks (sum of the cells in the aqueous and sediment phases) was calculated based on cell density measured in field samples prior to addition to the incubation flasks (F, 2 replicates), samples collected at the beginning of the incubation (T_0, 2 replicates from one incubation vessel), and samples collected at the end of each experiment (E, 3 replicates). Except for the experiment conducted in FDown during summer, the TCC in all experiments increased over the incubation time. As the results from F were more reproducible and the results from T_0 and E might have been influenced by the settling of microbes before sampling and sample transferring, and because for most chemicals, data from the early part of the incubation were used to determine k, the TCC calculated based on the measured bacterial cell density in F was used to calculate $k_{TCC,pH7}$.

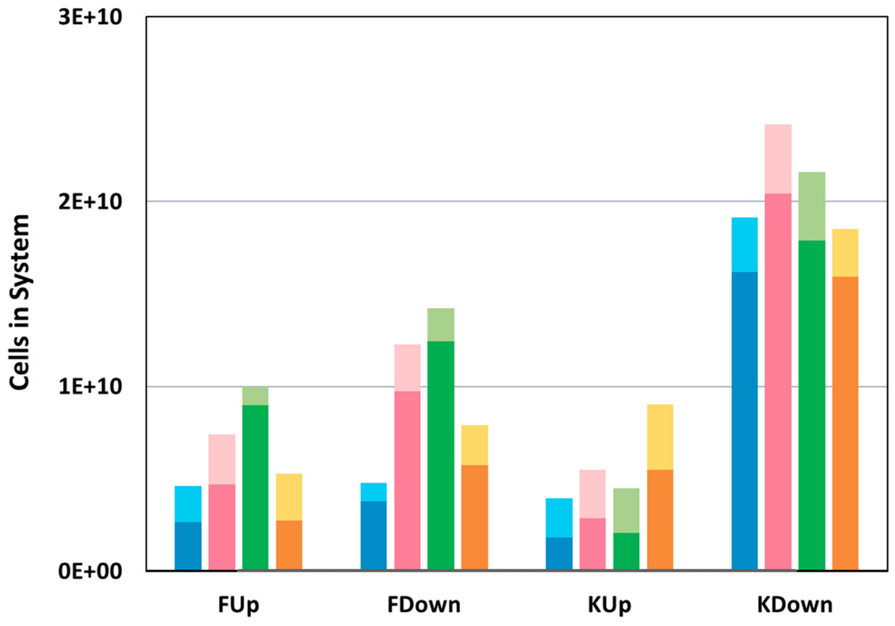

Figure S3. *Field total cell counts measured in water (shaded) and sediment (unshaded) in the experimental vessels (these values were used to calculate $k_{TCC,pH7}$). The results were from different seasonal experiments (Win.: winter, Spr.: spring, Sum.: summer, Aut.: autumn) at 4 different river sites (FUp, FDown, KUp, KDown).*

Table S4. *Total cell count (*10E+9, cells) in the experiment vessels (sum of the cells in the aqueous and sediment phases) calculated based on cell density measured in field samples prior to their addition to the experimental vessels (F) and samples collected at the beginning (T_0) and end of each experiment (E). The mean value of TCC with the standard error between replicates is shown in Table (T_0: standard error derived from analytical (not incubation) replicates, E: standard error derived from incubation replicates).*

River	FUp				FDown				KUp				KDown			
Season	Win.	Spr.	Sum.	Aut.	Win.	Spr.	Sum.	Aut.	Win.	Spr.	Sum.	Aut.	Win.	Spr.	Sum.	Aut.
F	4.6± 0.07	7.4± 0.2	10.0± 0.08	5.3± 0.2	4.8± 0.2	12.3± 0.4	14.2± 0.4	7.9± 0.3	3.9± 0.1	5.5± 0.2	4.5± 0.3	9.0± 0.3	19.2± 0.05	24.2± 0.2	21.6± 0.4	18.5± 0.07
T_0	7.8± 0.6	7.0± 0.1	11.5± 0.2	8.6± 0.1	14.7± 0.3	23.7± 1	17.9± 0.2	26.7± 1	9.7± 0.005	9.8± 0.4	4.8± 0.3	12.2± 0.5	29.4± 0.9	35.6± 1	25.6± 0.9	34.4± 0.8
E	4.7± 1	13.8± 2	12.2± 2	11.5± 1	14.3± 0.6	41.3± 0.7	12.3± 2	38.4± 2	23.7± 1	10.4± 0.9	7.0± 0.4	14.5± 3	20.3± 0.2	78.1± 3	23.9± 4	57.7± 2

S3. Quality control and quality assurance

S3.1 Environmental conditions

All experiments mirrored the environmental conditions (temperature, pH, DO, and conductivity) in the field well. DO in the water phase always increased to the saturated level (90% to 100%) within the first 5 hours of incubation and then became stable. All other environmental parameters were stable during incubation and reproducible between the 3 replicates. The standard deviation of the pH during incubation was < 0.2 in all experiments. The coefficient of variation of DO and conductivity was lower than 4% and 6%, respectively, during the incubation. The standard deviation of pH was < 0.11, and the coefficient of variation of DO and conductivity was < 6% and < 8%, respectively, between the 3 replicate test treatments at each time point. The results indicate that the systems were stable.

Table S5. *Mean of all pH values measured in the 3 replicate test incubations in each experiment.*

River	FUp				FDown				KUP				KDown			
Season	Win.	Spr.	Sum.	Aut.	Win.	Spr.	Sum.	Aut.	Win.	Spr.	Sum.	Aut.	Win.	Spr.	Sum.	Aut.
Mean pH	7.9	7.5	7.4	7.0	7.8	7.4	7.4	7.0	7.7	7.3	7.5	7.4	7.4	7.2	7.4	7.0

S3.2 Chemical analysis and experimental results

Sample preparation and chemical analysis were carried out as described by Tian et al.[2] using an ultrahigh-performance liquid chromatography system coupled to a Q Exactive HF Hybrid Quadrupole-Orbitrap mass spectrometer (UHPLC-Orbitrap-MS/MS, Thermo Fisher Scientific, San Jose, CA) with electrospray ionization (ESI). Data processing was carried out in Compound Discoverer 3.3. Filtered river water spiked with 1 µg L^{-1} test compounds and 10 µg L^{-1} internal standards was used as a quality control sample (QC) and measured every 6 samples. A 15-point matrix-matched calibration curve ranged from 1 ng L^{-1} to 10 µg L^{-1}. The calibration series was measured 2 times at the end of the sequence. The limit of detection (LOD) was defined as the concentration in the lowest calibration standard in which the test chemical was detected. The calibration curve was considered linear when the relative standard deviation (RSD) of the background corrected response factor was < 25%. The upper part of the calibration curve was always linear, while the lower part was not. Starting with the lowest standard (LOD), standards were successively removed from the calibration curve until the RSD fell below 25%. The concentration in the lowest calibration standard remaining within the calibration curve was set as the limit of quantification (LOQ). Only the data with a peak area higher than the LOQ were used for estimating k.

To correct for the intra-batch variability in processing multiple-batch UHPLC-Orbitrap-MS/MS data and to reduce the measurement errors, a drift correction based on the drift of QC samples analyzed between every 6 samples was conducted for each sequence using the R package *batchCorr*.[3] After QC correction, 99% of the identified test chemicals and internal standards had a coefficient of variation in the QC samples < 25% (Figure S4). Furthermore, in the samples from the test treatments the RSD of over 94% of the internal standards was < 25%. The good efficacy of the QC correction supports the feasibility of quantification without internal standard correction. Also, after QC correction none of the internal standards had a trend in signal (peak area) during the 10-day incubation. This indicates that there was no change in response factor as a result of a change in matrix effects over time.

96 out of 129 test compounds were identified and quantified in all experiments. The dissipation rate constants of those compounds were estimated using a Python algorithm with automatic recognition of attenuation curve slope changes over time (The algorithm is open source and available as a Python package "*chowclassifier*"). The schema for the Chow classifier is shown in Figure S5. The estimation of k is detailed in section S5. Using individual k values determined for the three test treatment replicates, we calculated the relative standard deviation (RSD) to assess the precision of the determination of k. It was < 30 % for > 77% of the degraded chemicals (Figure S4).

Table S6. *Number of chemicals degraded (Deg.), persistent (Per.) or for which data for k estimation was not available (N.A.) for each of the experiments.*

River	FUp				FDown				KUp				KDown			
Season	Win.	Spr.	Sum.	Aut.	Win.	Spr.	Sum.	Aut.	Win.	Spr.	Sum.	Aut.	Win.	Spr.	Sum.	Aut.
Deg.	61	67	72	66	63	60	54	46	51	65	58	60	60	65	55	62
Per.	26	12	21	28	28	30	32	47	29	14	31	30	27	19	31	31
N.A.	9	17	3	2	5	6	9	3	16	17	7	6	9	11	10	3

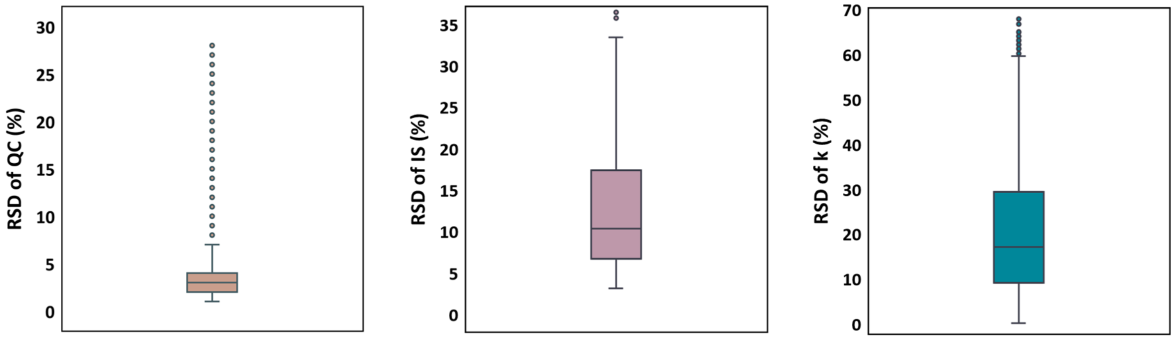

Figure S4. *RSD of the 96 targeted chemicals in the quality control (QC) samples after drift correction (left panel), of the 32 internal standards in all samples after drift correction (middle panel), and of k (right panel).*

S3.3 Python algorithm (Chowclassifier) for recognizing the breakpoint in biphasic kinetics

The Chow test was first derived by Gregory Chow[4] in 1960 and later by Franklin Fisher.[5] The algorithm that was developed for this paper performs one or several Chow tests on a time-indexed dataset to determine if a breakpoint in the time trends is statistically significant at a chosen confidence level α. For each breakpoint tested, the dataset is modelled as $y_i = a_1 x_i + b_1$ for the data points x_i before the breakpoint, and $y_i = a_2 x_i + b_2$ for the data points x_i after the breakpoint.

The significance of a breakpoint is tested under the null hypothesis that $a_1 = a_2$ and $b_1 = b_2$ (and the alternative hypothesis that $a_1 \neq a_2$ and $b_1 \neq b_2$) using the statistic $Z = \frac{S_C-(S_1+S_2)}{S_1+S_2} \cdot \frac{N_1+N_2-2k}{k}$, where k=2 is the total number of parameters, S_C is the sum of squared residuals of the regression on the full time series, S_1, S_2 are the sums of squared residuals of the regression on the first, and respectively, the second half of the time series and N_1, N_2 are the number of observation for each half.

The statistic Z follows an F-distribution with k and $N_1 + N_2 - 2k$ degrees of freedom[2]. If multiple breakpoints are tested, a Bonferroni correction is applied (i.e., level tested in the F-distribution is $\frac{\alpha}{m}$, where m is the number of breakpoints tested).

Following the result of the Chow test, a linear model is applied to the set (if insignificant breakpoint) or to each of the two sets separately (if significant breakpoint) and the data is classified in one of the 14 categories in Table S7 and Figure S5.

Table S7. *Description of the categories classified based on the result of the Chow test.*

**Breakpoint**	**Category**	**Description**
No	N	non-significant overall trend
No	I	significant increasing overall trend
No	D	significant decreasing overall trend
Yes	NN	non-significant trend on set1 and non-significant trend on set2
Yes	NI	non-significant trend on set1 and significant increasing trend on set2
Yes	ND	non-significant trend on set1 and significant decreasing trend on set2
Yes	IN	significant increasing trend on set1 and non-significant trend on set2
Yes	ID	significant increasing trend on set1 and significant decreasing trend on set2

Yes	iI	significant increasing trend on both set1 and set2 with greater increase in set2
Yes	Ii	significant increasing trend on both set1 and set2 with greater increase in set1
Yes	DN	significant decreasing trend on set1 and non-significant trend on set2
Yes	DI	significant decreasing trend on set1 and significant increasing trend on set2
Yes	dD	significant decreasing trend on both set1 and set2 with greater decrease in set2
Yes	Dd	significant decreasing trend on both set1 and set2 with greater decrease in set1

where set1 and set2 indicate respectively the data before the breakpoint and after.

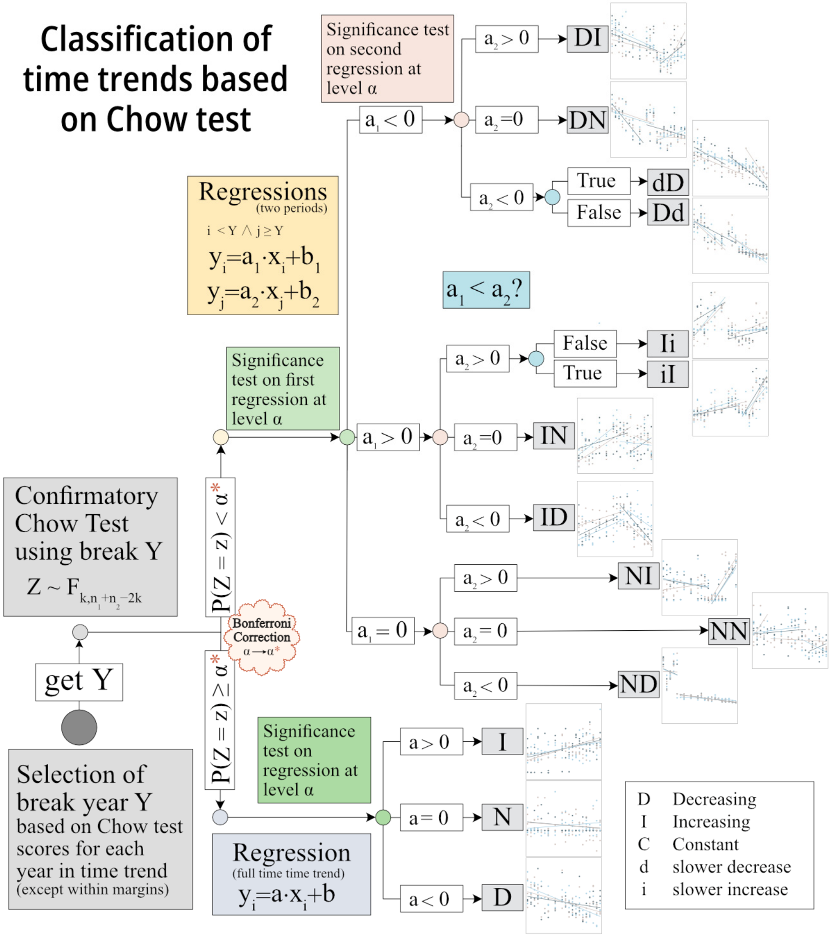

Figure S5. *The schema for the chowclassifier. Y = ln (peak area/peak area*$_0$*), x = time, and a is the rate constant k.*

S4. Abiotic dissipation

The abiotic controls were meant to distinguish dissipation via biodegradation from dissipation via abiotic attenuation (hydrolysis and sorption). There were five chemicals that showed dissipation in the hydrolysis control (ethylparaben, methyl 4-hydroxybenzoate, nicotinamide, propyl 4-hydroxybenzoate, and triethyl citrate). Methyl 4-hydroxybenzoate only dissipated in the Knivstaån experiments, and triethyl citrate only dissipated in the Vitsån experiments. Propyl_4-hydroxybenzoate dissipated in both rivers but only in the spring experiments.

Before the start of the experiment, 16 out of 96 quantified compounds (4-(4-nitrobenzyl)-pyridine, amisulpride, citalopram, climbazole, decylamine, dibenzepin, flecainide, fluoxetine, levamisole, nicotinamide, propranolol, ranitidine, tamsulosin, trimethoprim, venlafaxine, and zolpidem) were significantly sorbed to the sediment (higher than 2-fold difference in peak area between hydrolysis control and SC). This was consistent across seasonal experiments at different locations (Supplemental Dataset S1).

During the experiments, between 10 and 25 out of 96 compounds showed a first-order dissipation with a slope significantly different from 0 in SC. The ratio between the dissipation rate constants in the test treatments and the SC is shown in Table S8. Out of 1402 cases, only 81 cases had a dissipation rate constant in the SC that was not at least 2.5 times smaller than k derived from the test treatments. Five further compounds (chlorthalidone, hydrochlorothiazide, hydroxy bupropion, imidacloprid, oxazepam) that had dissipation in the SCs were classified as persistent after sorption subtraction. Overall, dissipation in the SC had a small effect on the experimental results.

Dissipation in the SC during the experiments in excess of that observed in the hydrolysis control might be due to: i) abiotic reactions facilitated by the presence of the sediment; ii) sorption; iii) residual biodegradation as a result of incomplete sterilization. In the first two cases a SC correction of the test treatments is indicated, while in the third case an SC correction should not be conducted. We could not ascertain which of these three phenomena was responsible for the dissipation in our experiments. Our decision to correct all of the test treatment data for dissipation in SC may have resulted in an underestimation of k in a few cases if the dissipation in SC was caused by phenomenon iii).

Table S8. *Ratio between dissipation rate constants in test treatments and the sorption controls (SC) for chemicals showing a significant k and dissipation in SC in at least one experiment. Values marked with an asterisk indicate that the ratio is less than 2.5. Empty cells indicate that no significant dissipation was observed in the SC. For elaboration of the compound name abbreviations see Table S1.*

River	FUp				FDown				KUp				KDown			
Season	Win.	Spr.	Sum.	Aut.	Win.	Spr.	Sum.	Aut.	Win.	Spr.	Sum.	Aut.	Win.	Spr.	Sum.	Aut.
5MB		2.5	7.1	1.5*	1.6*	1.7*	1.6*	3.6	5.4	1.7*	1.6*	1.9*		4.9		
ABA	5.6	13.1	13.8	3.0	9.3	7.6	9.4	4.0	4.6	4.7	5.2	8.6	5.9	5.3	8.7	
ATP						1.8*	4.2								4.5	
AMI		1.6*		3.0	1.8*	1.5*		4.9			1.1*	1.1*				
ALA										2.1*						
ATE		4.3	3.4	2.8		2.8	3.0	2.3*					3.9	2.8	1.8*	2.1*
BIS		7.5			8.1	2.2*							1.6*	4.5		2.0*
BTZ		4.9	3.0	2.1*	2.6	2.5	2.6	2.0*	6.7	3.6				4.4		
CAR					2.2*	2.2*										
CIL		130.7	42.4			47.7	42.2	37.5		29.6		7.3		77.0	70.7	55.7
CIT			6.8	1.9*												
DEC	12.3		7.9	11.0	13.3		20.6	24.2	2.8	12.0	4.9	23.9	4.2		10.0	
DIB			1.6*	1.4*	1.4*	1.4*										
LAM	6.3	7.7				4.4										
LAS						1.4*	1.3*	9.5					1.4*	1.9*		
LID					1.9*	1.1*	0.6*									
LIN			4.3	2.3*												
LOS										15.0		5.5				
NEO														2.0*		2.3*
NOV										1.9*		2.4*				
OXP		2.0*		1.5*	1.6*	1.3*	1.2*			1.7*	1.2*	1.3*	1.2*	1.6*		
PAR	10.4			7.3												
PGL		9.5	11.7							10.8		8.7		10.2		
PPC	4.3	8.7	6.5	5.7		1.5*	4.3	6.6	1.3*			6.6		3.6		
PPN						1.6*				15.0		5.5				
RAN	2.7	1.4*	1.2*		5.7	1.5*	2.6	3.2	2.5	2.0*	10.3	3.0	4.8	2.1*	1.4*	1.3*
RUF		8.6	3.8	5.4	6.6	3.3	4.4			8.3	8.4	7.5	5.4	6.5	2.6	6.0
SOT									1.9*	3.7		4.0	1.4*	2.4*		1.9*
SMP									11.4	6.9						
STZ	7.6	11.0	9.5	9.1			21.4	3.3								
TRA									1.3*	1.4*	1.1*	9.2				
TRC	1.8*	2.0*		1.4*	1.7*	0.7*	1.1*	1.7*					1.6*	1.8*	1.1*	1.4*
TMP		3.4			1.5*	1.7*										
TAE		12.9								4.6		8.1		14.0	8.1	7.4

S5. Biodegradation kinetics of quantified test compounds

The peak area (A) of each test compound at each time point was normalized to its peak area at t=0 (A_0). The natural logarithm of the normalized peak areas derived from SC was subtracted from that in test treatment at each time point, and the resulting data were used to quantify k. All data used to estimate biodegradation kinetics were SC corrected. All data derived from the 3 test incubations were used together for rate constant quantification by the Chowclassifier script (Section S3.3). The quantified k values were used to compare biodegradation between seasons. We also calculated k using data from each test-treatment replicate to assess the reproducibility of the incubation test (Figure S4) and the significance of the differences in k between seasons (Figure S8). When k was significantly different from 0 (p-value < 0.05), it was considered valid. All of the estimated k were used for comparison regardless of whether or not they were significantly different from 0 to obtain more information (Figure S7). When the rate constant was < 0.0001 or a negative k was not significantly different from 0 (p-value > 0.05), it was set to 0.0001. Only 1.7% of estimated k-values (24 out of 1402) displayed a significant increase in concentration over time ($p < 0.05$), and half of the chemicals with a negative k tended to increase over time only during the first two days of incubation. The negative k was judged to arise from uncertainty in the sorption correction and chemicals with negative k were excluded from the seasonal comparison. To minimize the impact of possible fluctuations within the first incubation day on the regression analysis, time points during the first day were not used in estimating k unless the compounds degraded rapidly to below LOQ within 1 day. Only the initial k was considered environmentally relevant when biphasic kinetics was observed. k was considered not valid when there were less than 5 data points above LOQ. The obtained biodegradation kinetic parameters are listed in Supplemental Dataset S1.

The impact of the sorption of compounds was accounted for by dividing *k observed* by f_{Dis} (Supplemental Dataset S1):

$$k = k\ observed * \frac{1}{f_{Dis}}$$

The uncertainty of k was significantly increased after the sorption correction for amisulpride, dibenzepin, and levamisole, we thus excluded them from seasonal comparisons in this study.

To investigate if the changes in neutral fraction (f_N) and TCC are associated with the seasonality of k, we have normalized k to standard conditions in 2 steps, first to pH 7, then to a reference TCC in the natural environment. We calculated the normalized k obtained at each step as follows.

Firstly, k was normalized to pH 7 to obtain k_{pH7}. The neutral fraction (f_N) was predicted using MarvinSketch 23.3 software from ChemAxon. We only did the pH correction for the ionizable chemicals. The observed k was corrected to a reference pH of 7 by multiplying k by the ratio of the f_N at pH 7 to that at the mean pH in the test incubation (Table S5) to obtain k_{pH7}. When f_N was <0.001 we assumed that there was no further influence of pH on k and no correction was made. The f_N of each compound and k_{pH7} are also listed in Supplemental Dataset S1 for the different experiments.

$$k_{pH7} = k * \frac{f_N of\ compounds\ at\ pH\ 7}{f_N\ of\ compounds\ at\ mean\ test\ pH}$$

In the second step, k_{pH7} was normalized to a reference TCC to obtain $k_{TCC,pH7}$. The reference level of TCC was calculated based on the global average of cell densities in surface waters, ~10^6 cells mL^{-1} [6], which was assumed to correspond to 10^9 cells in one laboratory vessel. The

resulting $k_{TCC,pH7}$ values are listed in Supplemental Dataset S1, and the TCC data are listed in Table S4.

$$k_{TCC,pH7} = k_{pH7} * \frac{10^9 cells\ (reference\ TCC)}{TCC}$$

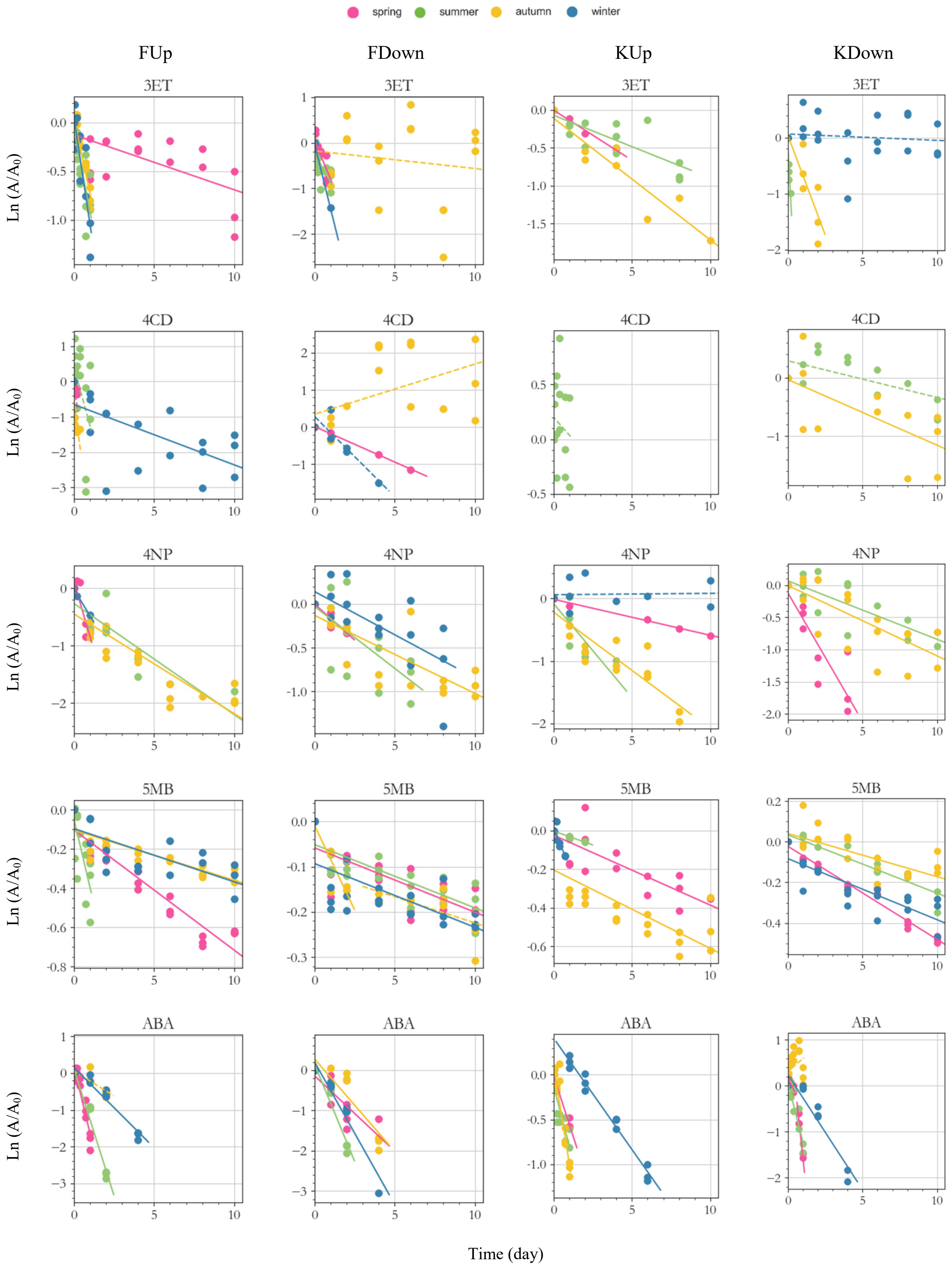

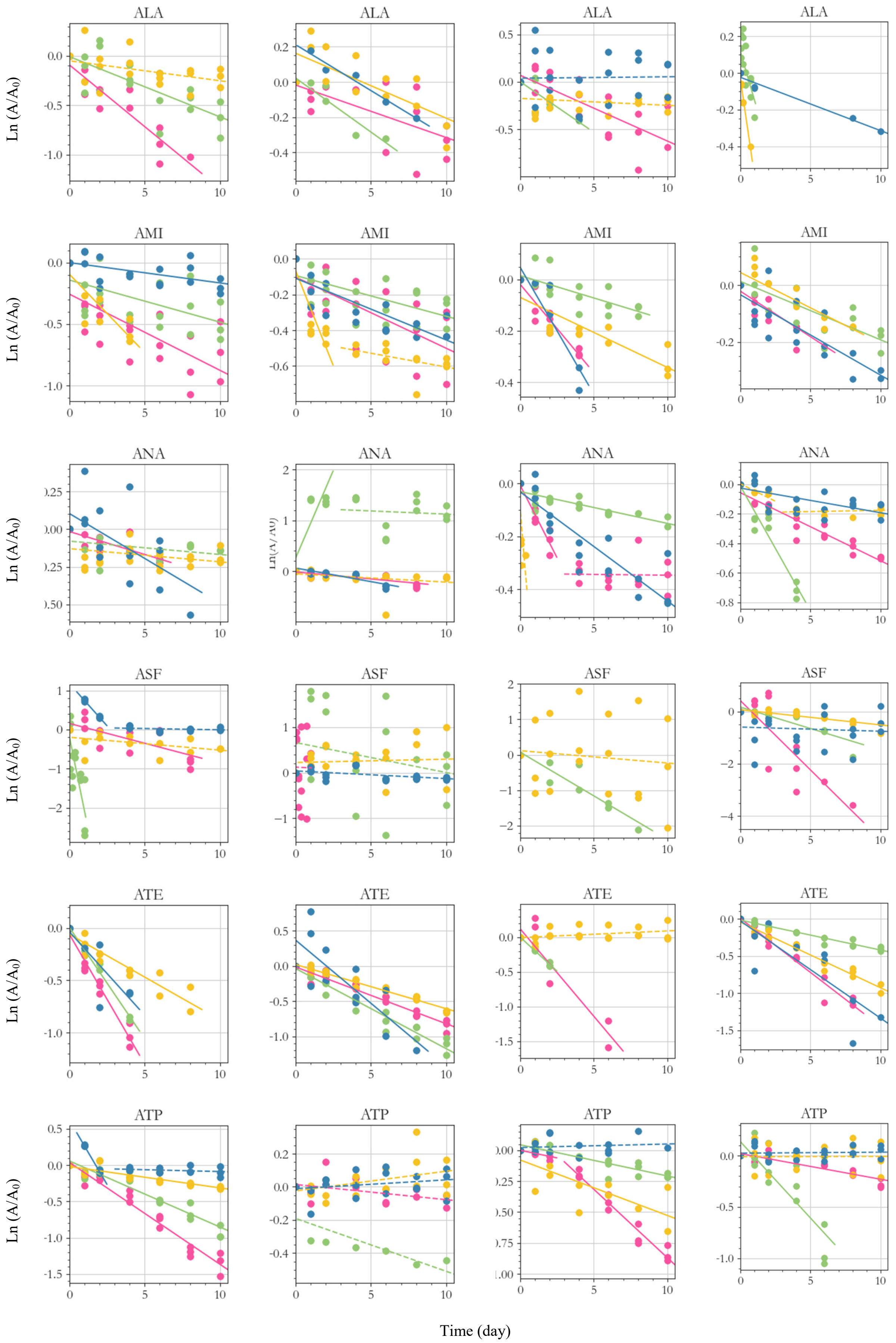

ALA
ALA
ALA
ALA
AMI
AMI
AMI
AMI
ANA
ANA
ANA
ANA
ASF
ASF
ASF
ASF
ATE
ATE
ATE
ATE
ATP
ATP
ATP
ATP
Ln (A/A$_0$)
Time (day)

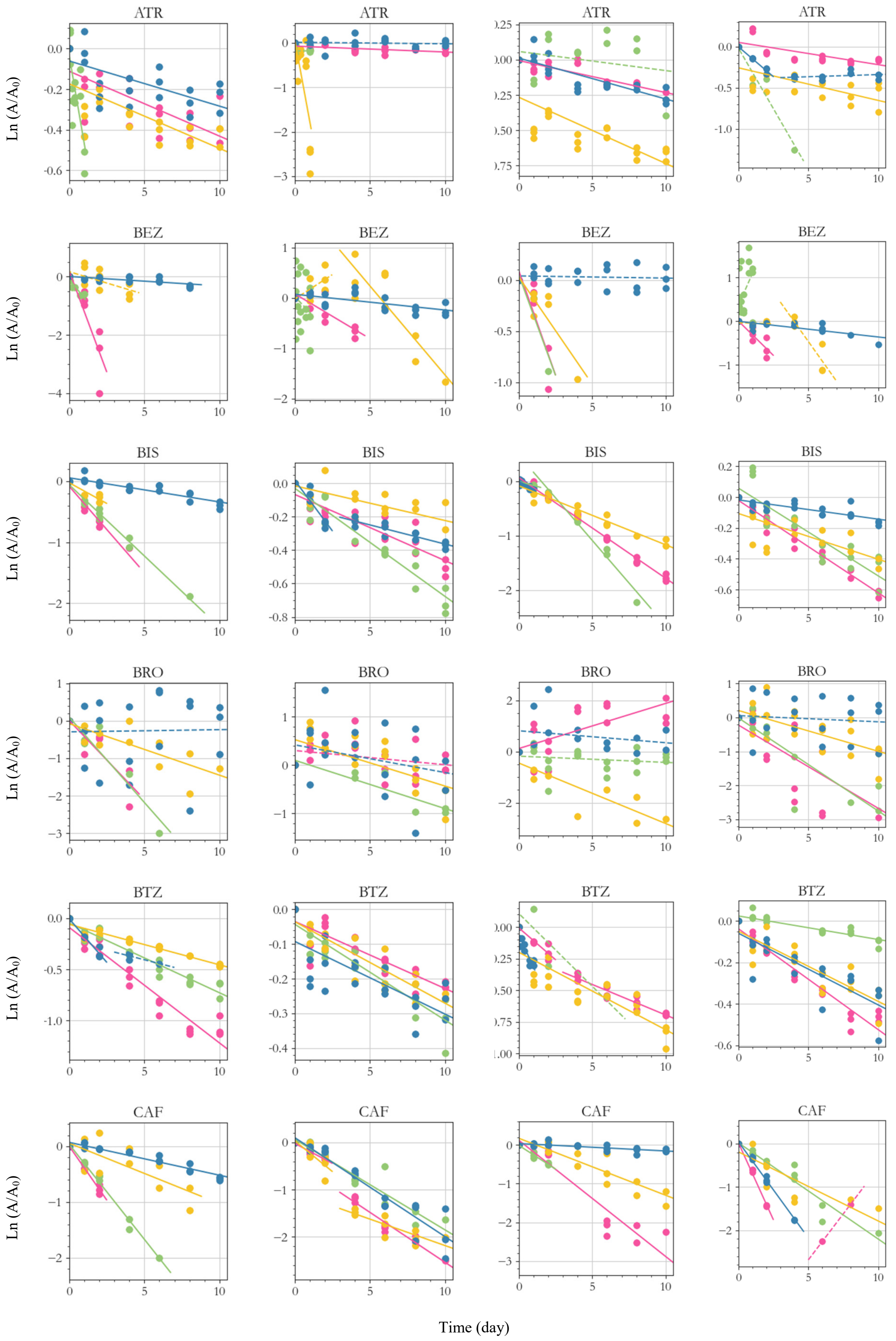
ATR
BEZ
BIS
BRO
BTZ
CAF
Ln (A/A0)
Time (day)

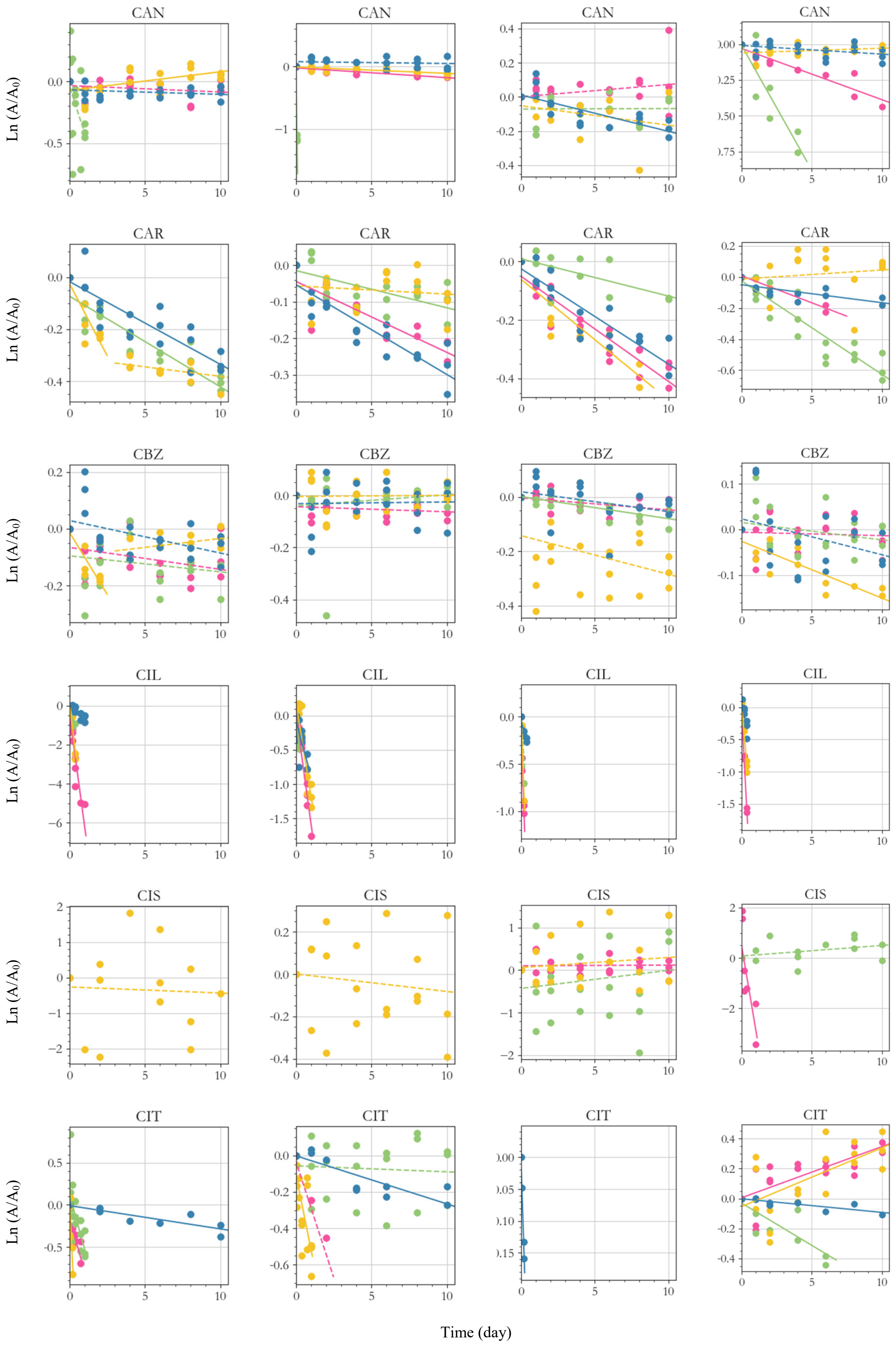
CAN
CAR
CBZ
CIL
CIS
CIT
Ln (A/A0)
Time (day)

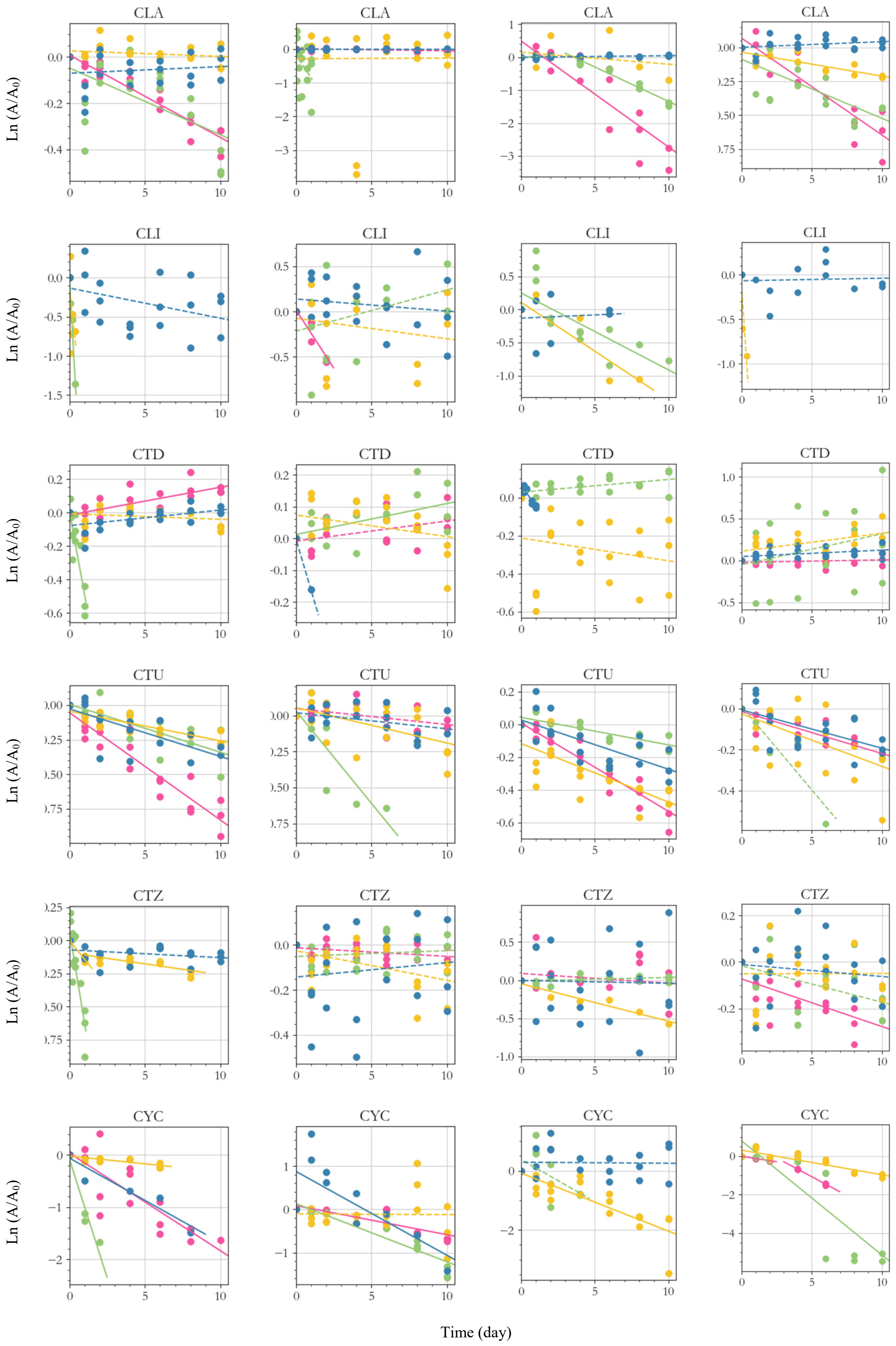
CLA
CLI
CTD
CTU
CTZ
CYC
Ln (A/A0)
Time (day)

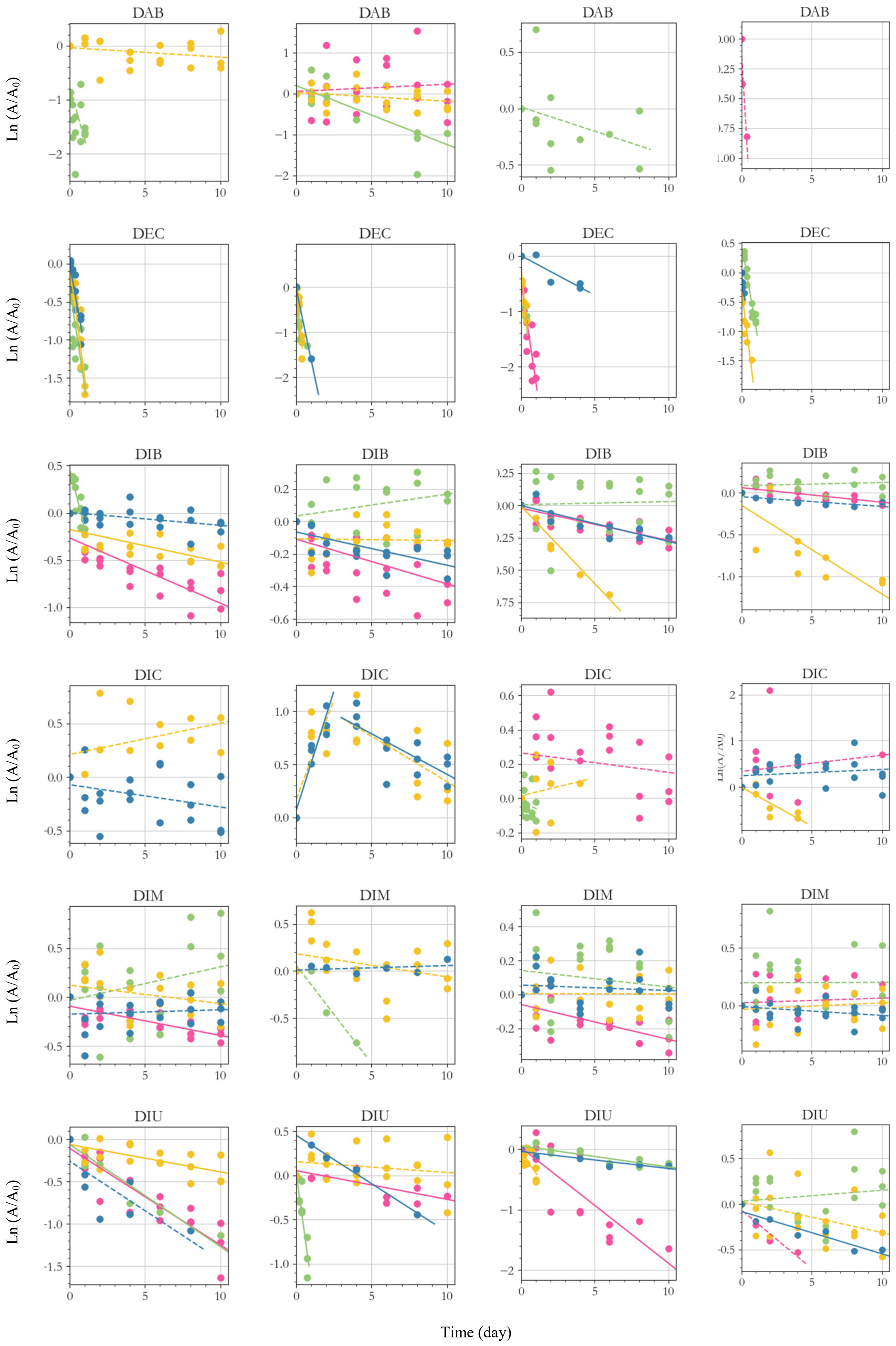
DAB
DEC
DIB
DIC
DIM
DIU
Ln (A/A$_0$)
Time (day)

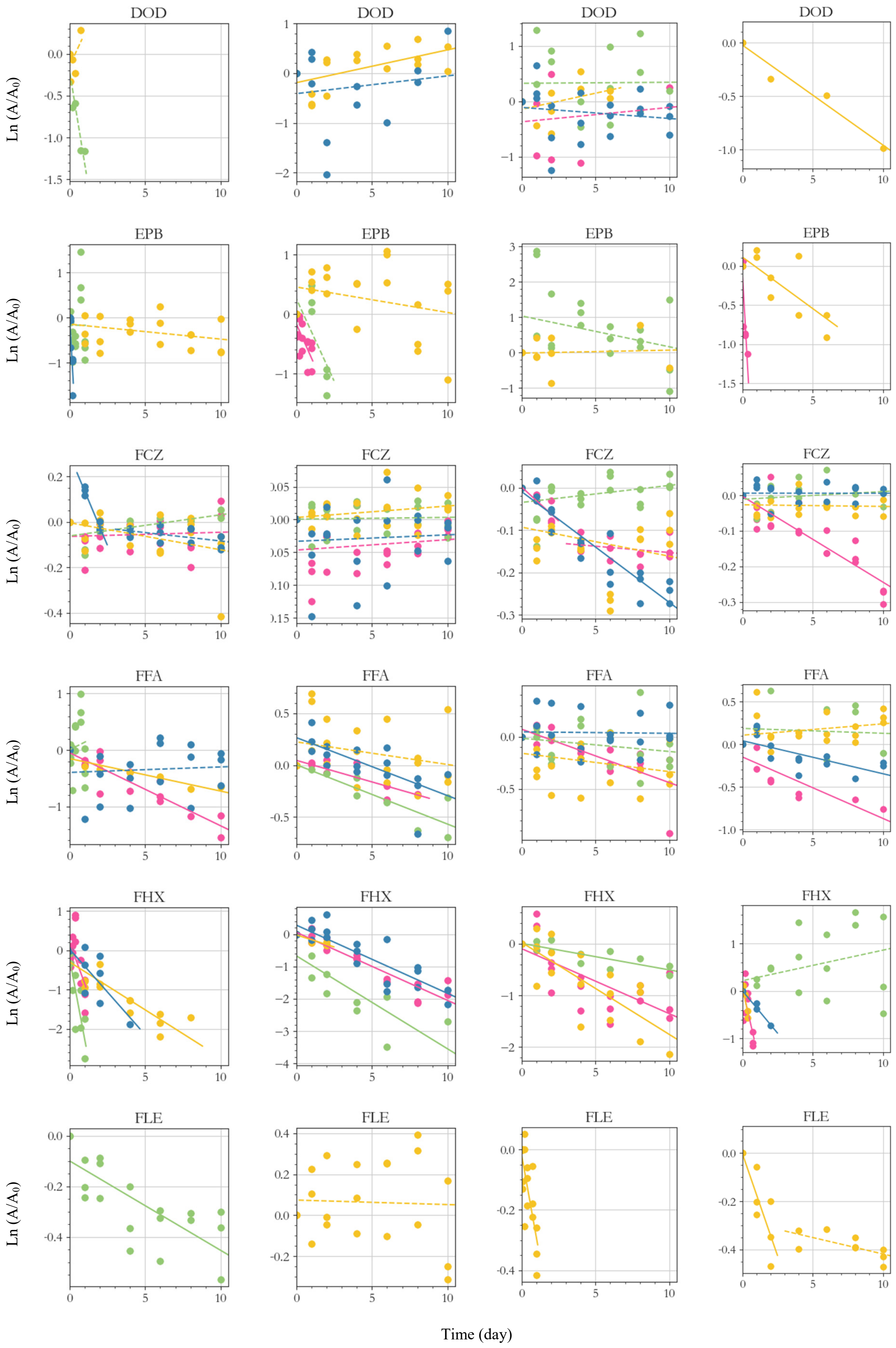
DOD
DOD
DOD
DOD
EPB
EPB
EPB
EPB
FCZ
FCZ
FCZ
FCZ
FFA
FFA
FFA
FFA
FHX
FHX
FHX
FHX
FLE
FLE
FLE
FLE
Ln (A/A$_0$)
Time (day)

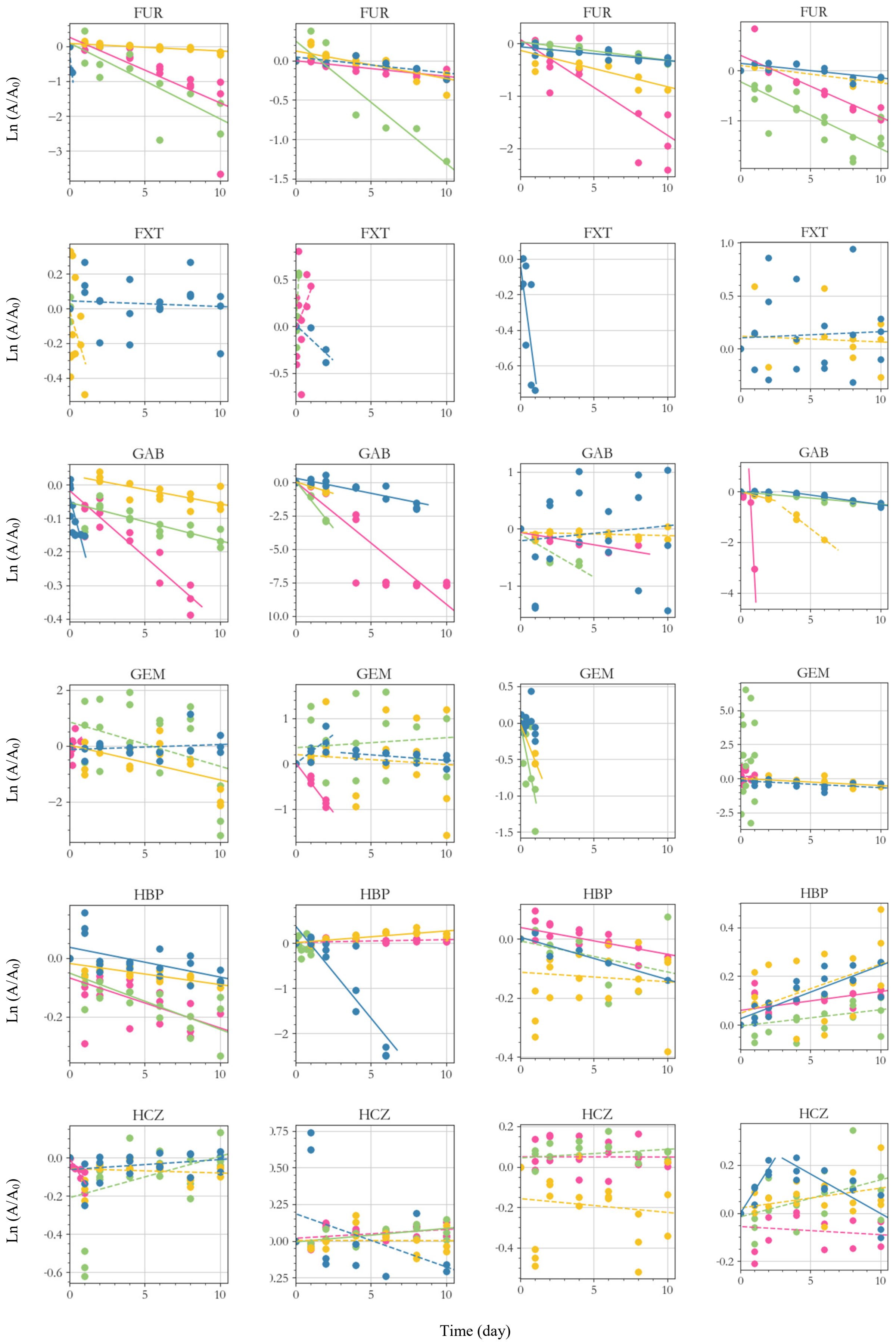
FUR
FUR
FUR
FUR
FXT
FXT
FXT
FXT
GAB
GAB
GAB
GAB
GEM
GEM
GEM
GEM
HBP
HBP
HBP
HBP
HCZ
HCZ
HCZ
HCZ
Ln (A/A0)
Time (day)

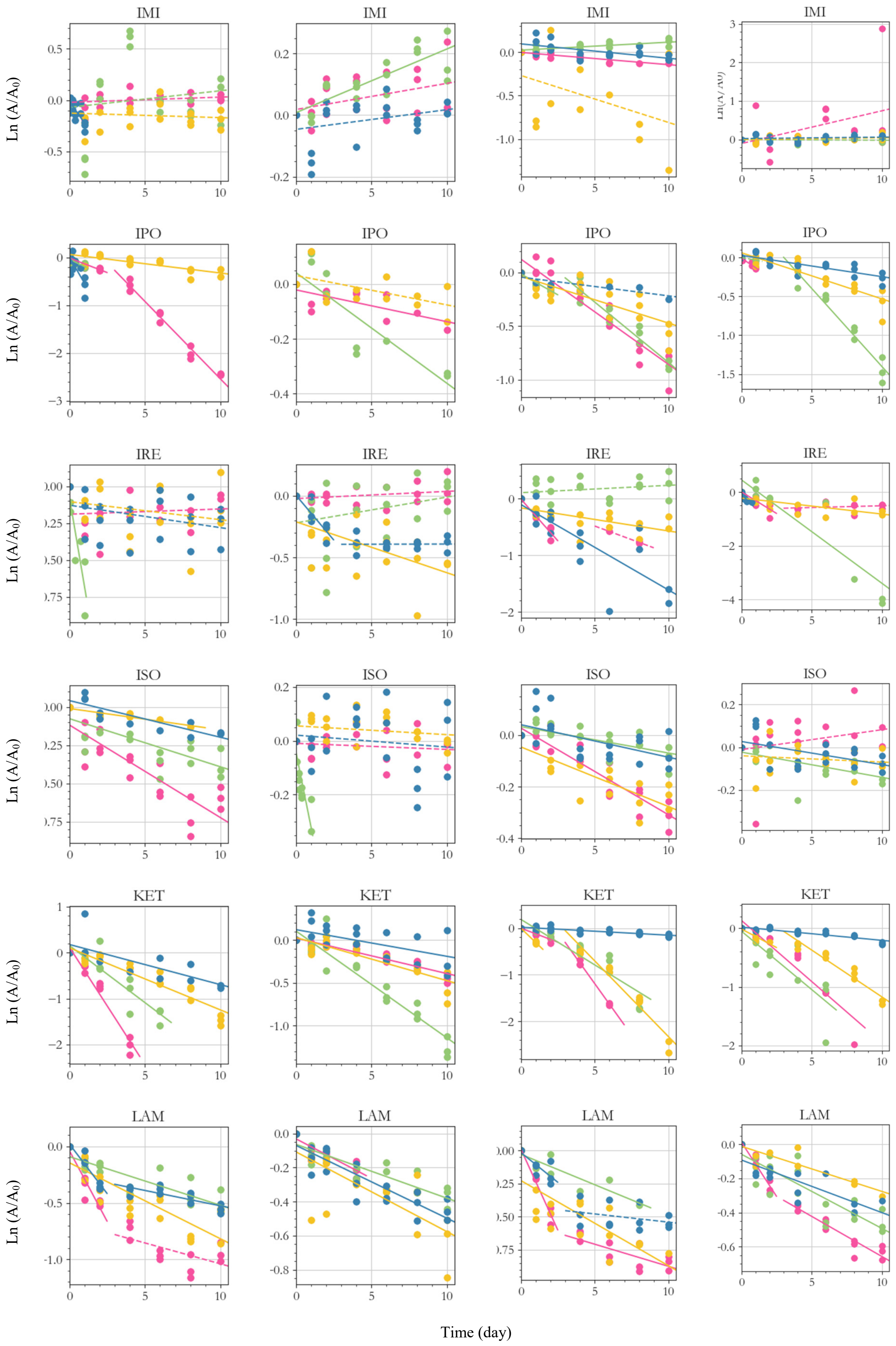
IMI
IPO
IRE
ISO
KET
LAM
Ln (A/A0)
Time (day)

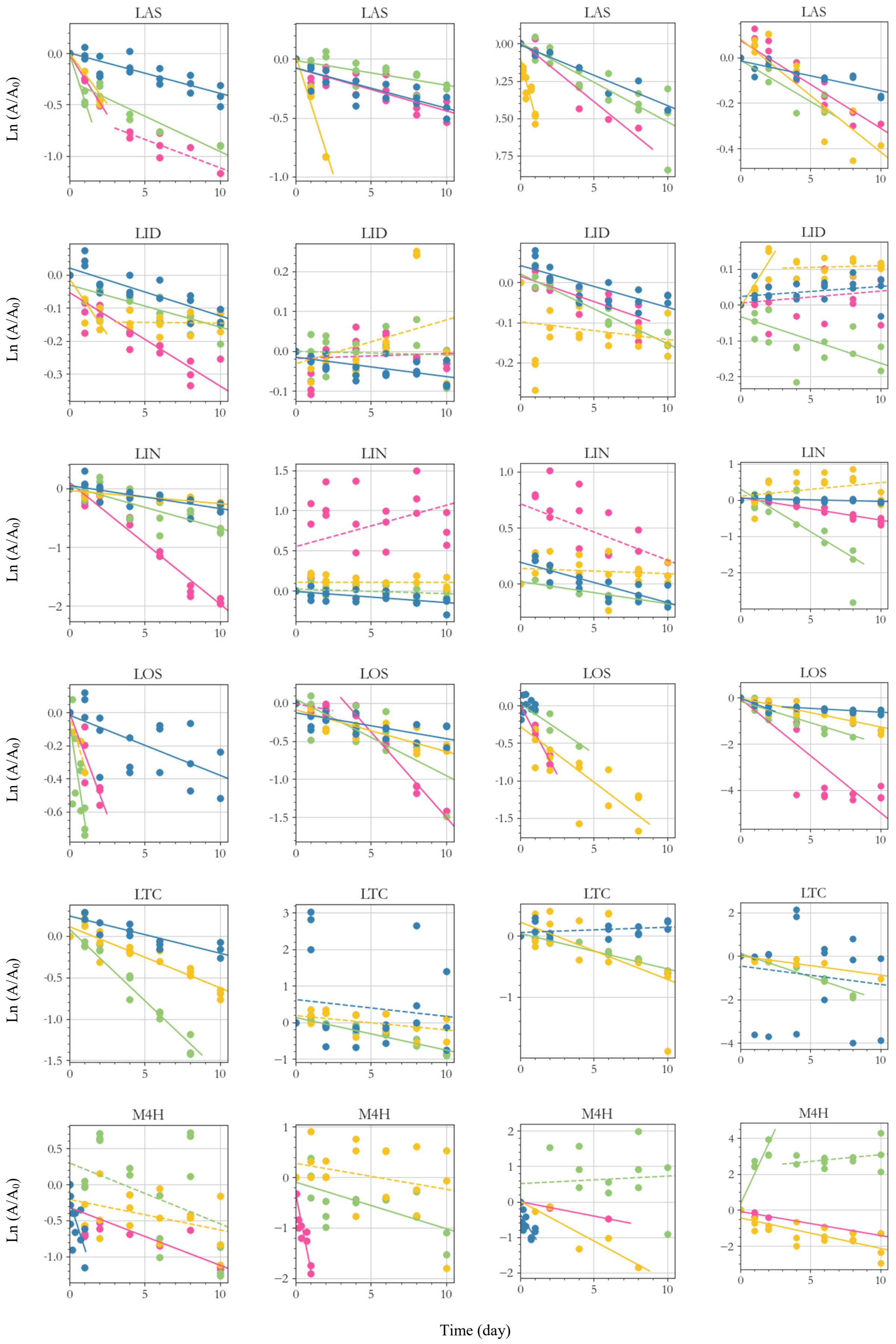
LAS
LAS
LAS
LAS
LID
LID
LID
LID
LIN
LIN
LIN
LIN
LOS
LOS
LOS
LOS
LTC
LTC
LTC
LTC
M4H
M4H
M4H
M4H
Ln (A/A0)
Time (day)

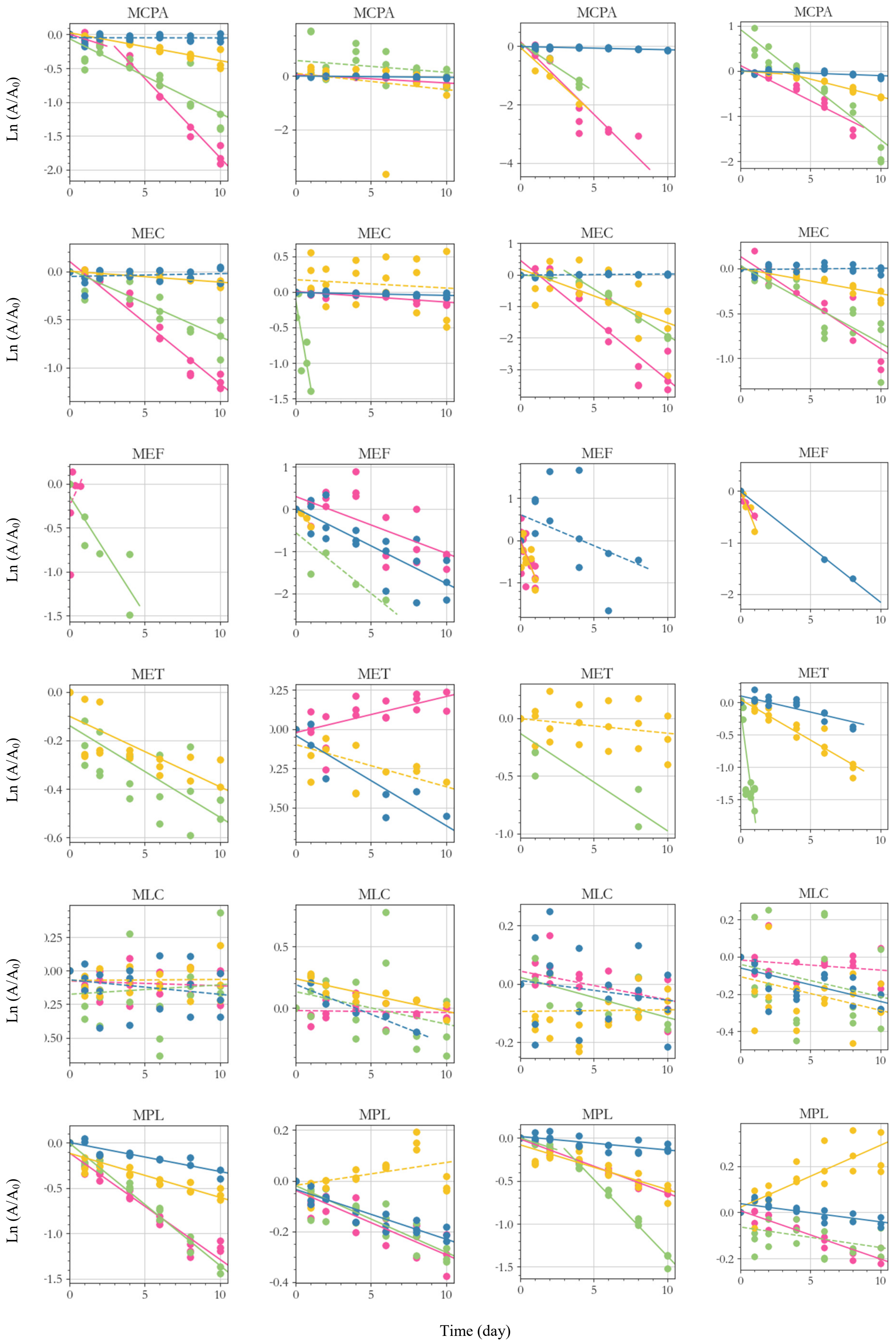
MCPA
MCPA
MCPA
MCPA
MEC
MEC
MEC
MEC
MEF
MEF
MEF
MEF
MET
MET
MET
MET
MLC
MLC
MLC
MLC
MPL
MPL
MPL
MPL
Ln (A/A0)
Time (day)

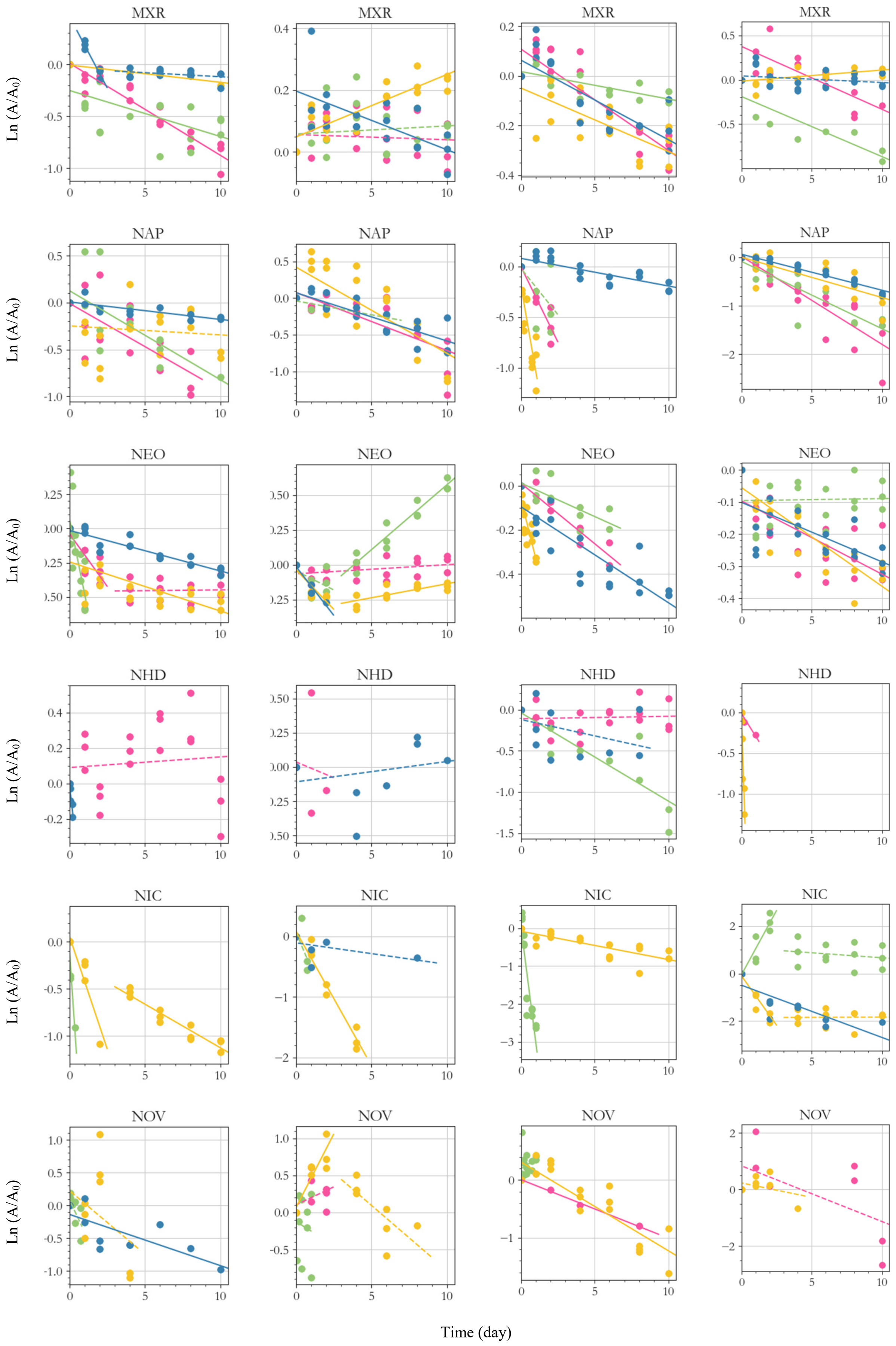
MXR
NAP
NEO
NHD
NIC
NOV
Ln (A/A0)
Time (day)

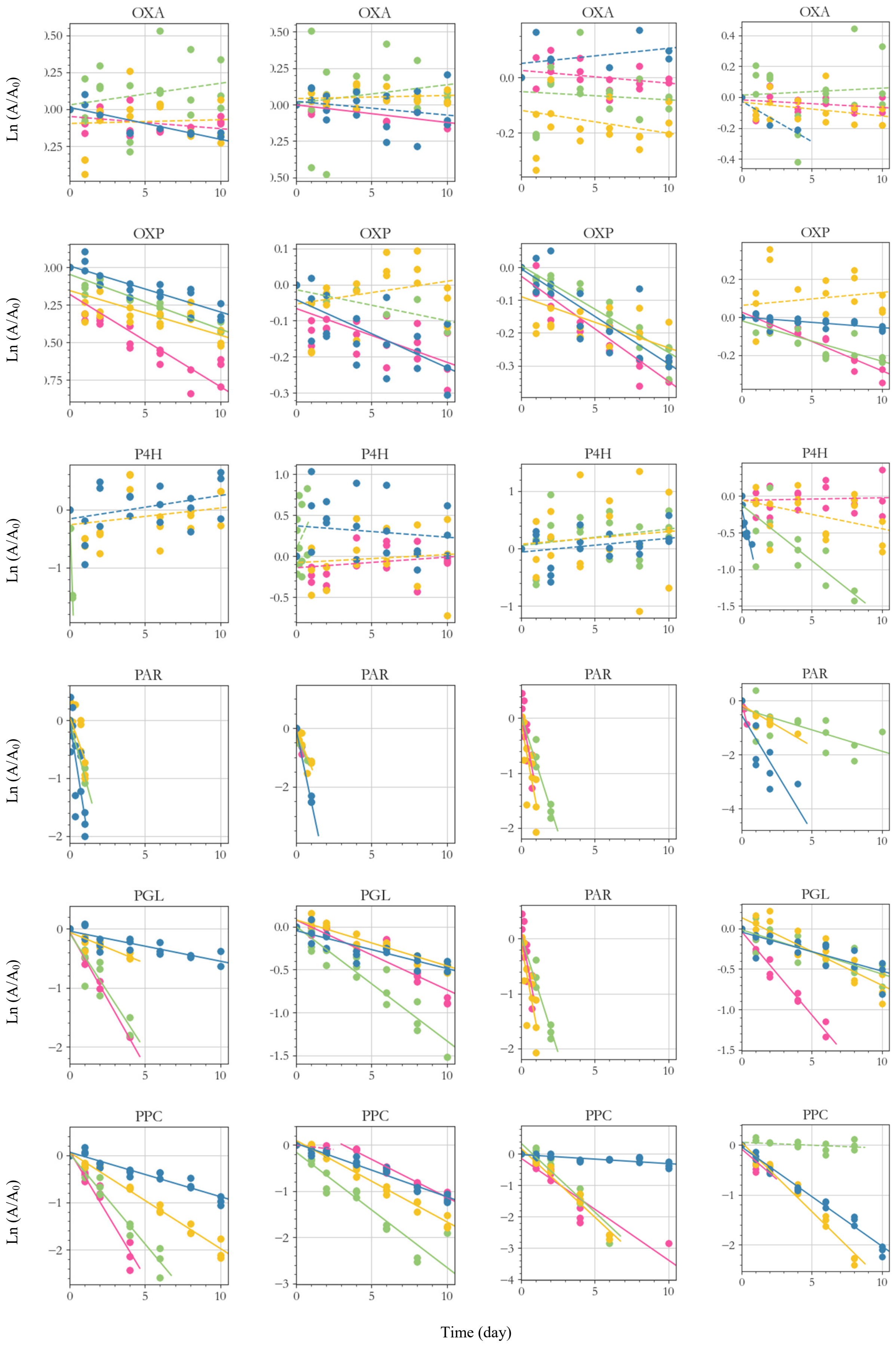
OXA
OXA
OXA
OXA
OXP
OXP
OXP
OXP
P4H
P4H
P4H
P4H
PAR
PAR
PAR
PAR
PGL
PGL
PAR
PGL
PPC
PPC
PPC
PPC
Ln (A/A0)
Time (day)

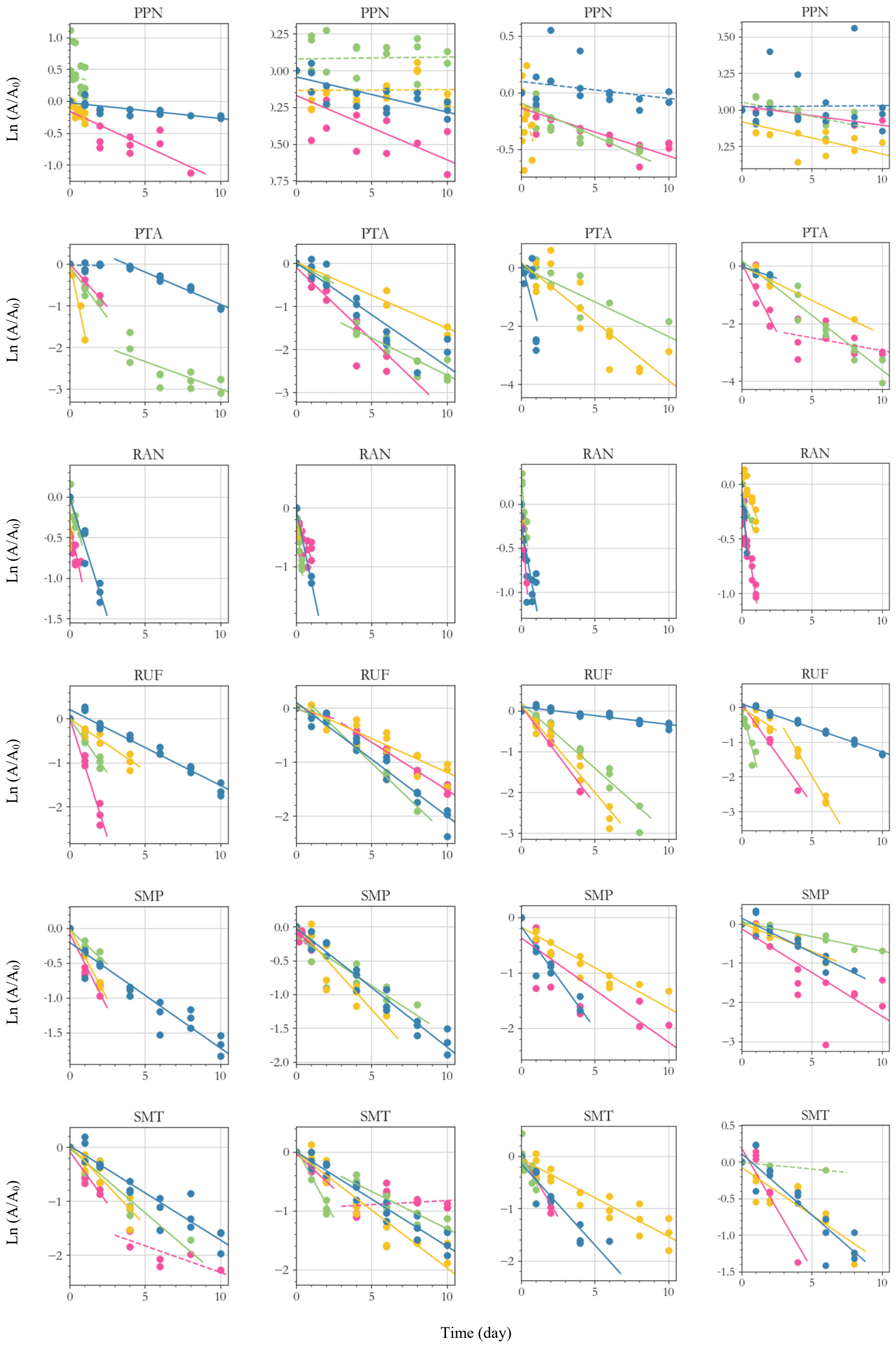
PPN
PTA
RAN
RUF
SMP
SMT
Ln (A/A0)
Time (day)

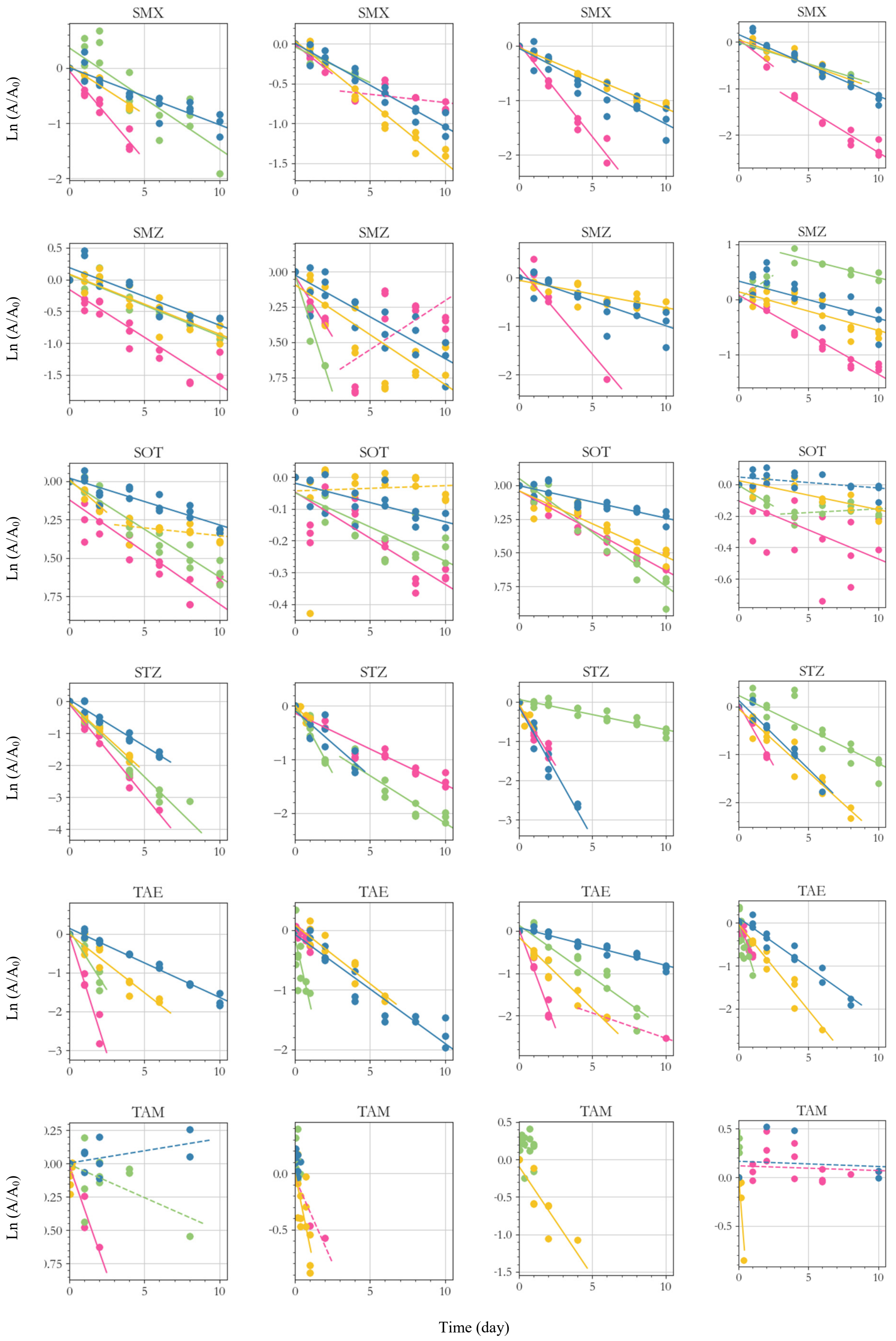
SMX
SMZ
SOT
STZ
TAE
TAM
Ln (A/A₀)
Time (day)

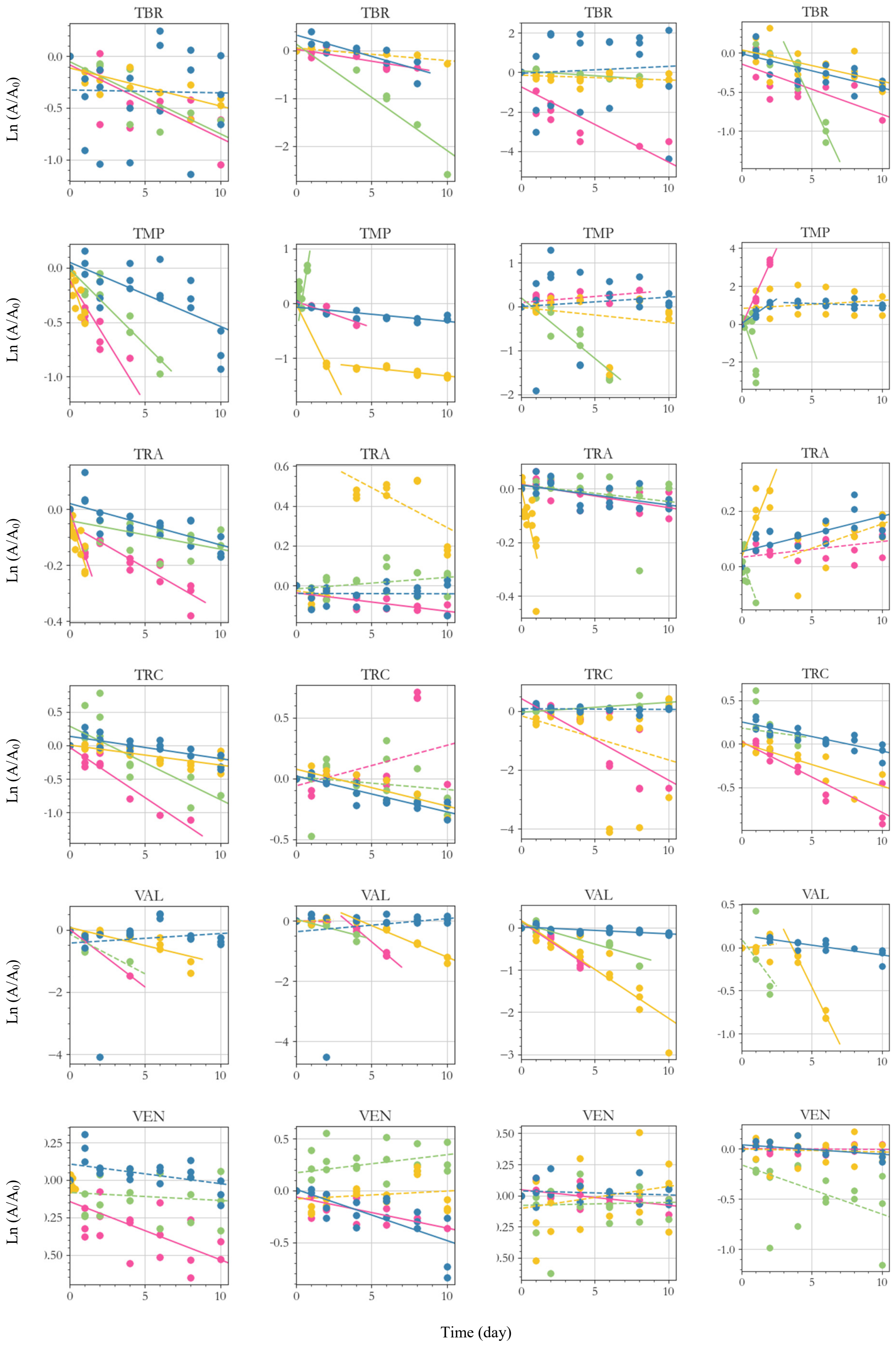
TBR
TMP
TRA
TRC
VAL
VEN
Ln (A/A0)
Time (day)

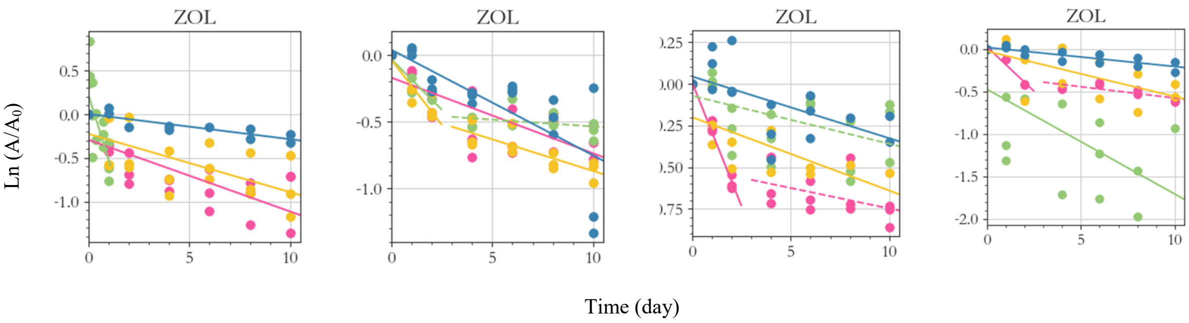

Figure S6. *Biodegradation kinetics of quantified test chemicals. A dashed line indicates that the 99% confidence interval of the slope intersected 0. The results were from different seasonal experiments (Win.: winter, Spr.: spring, Sum.: summer, Aut.: autumn) at 4 different river sites (FUp, FDown, KUp, KDown).*

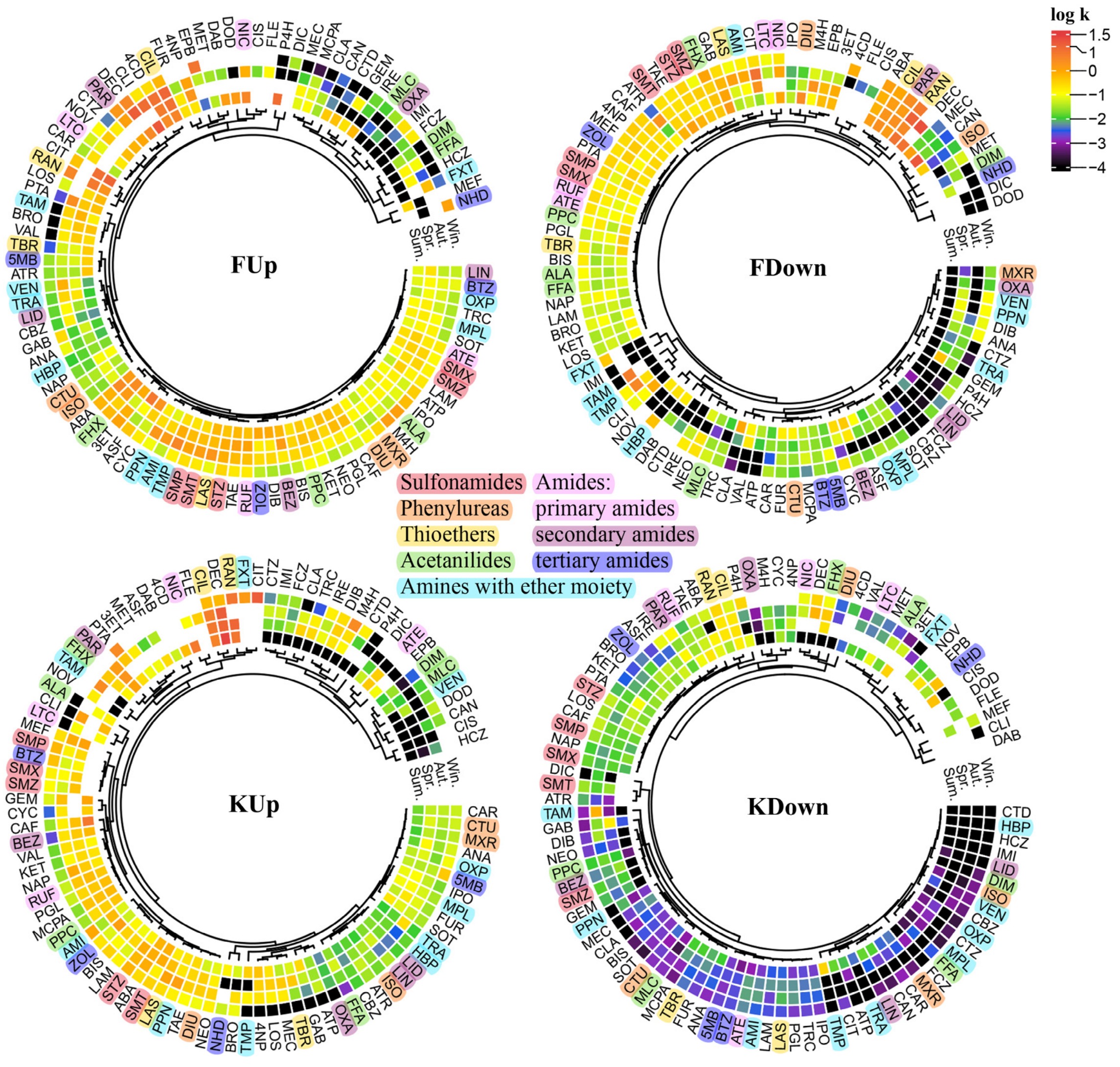

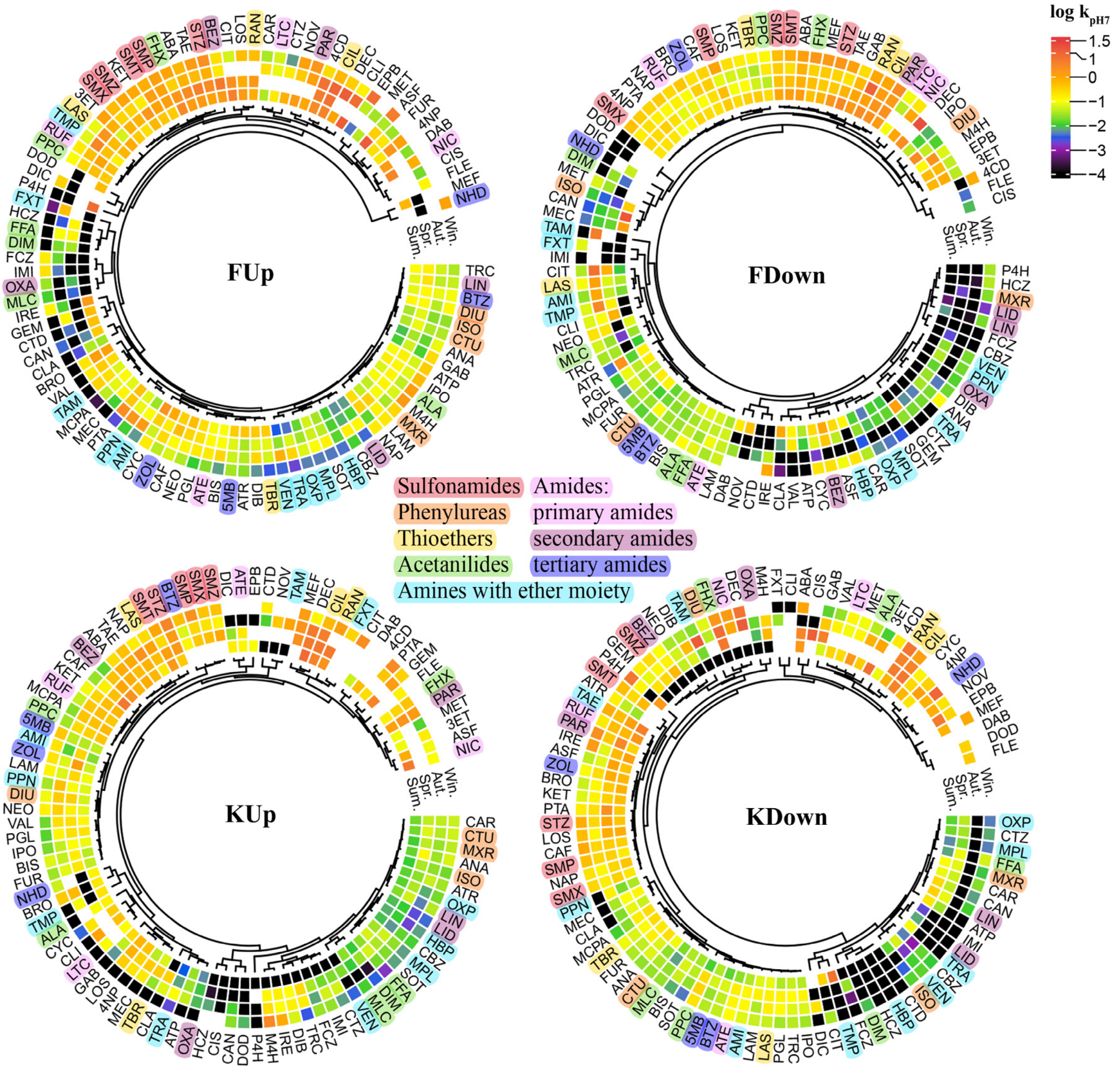
log k_{pH7}
1.5
1
0
−1
−2
−3
−4
FUp
FDown
KUp
KDown
Win.
Aut.
Spr.
Sum.
Sulfonamides
Phenylureas
Thioethers
Acetanilides
Amines with ether moiety
Amides:
primary amides
secondary amides
tertiary amides

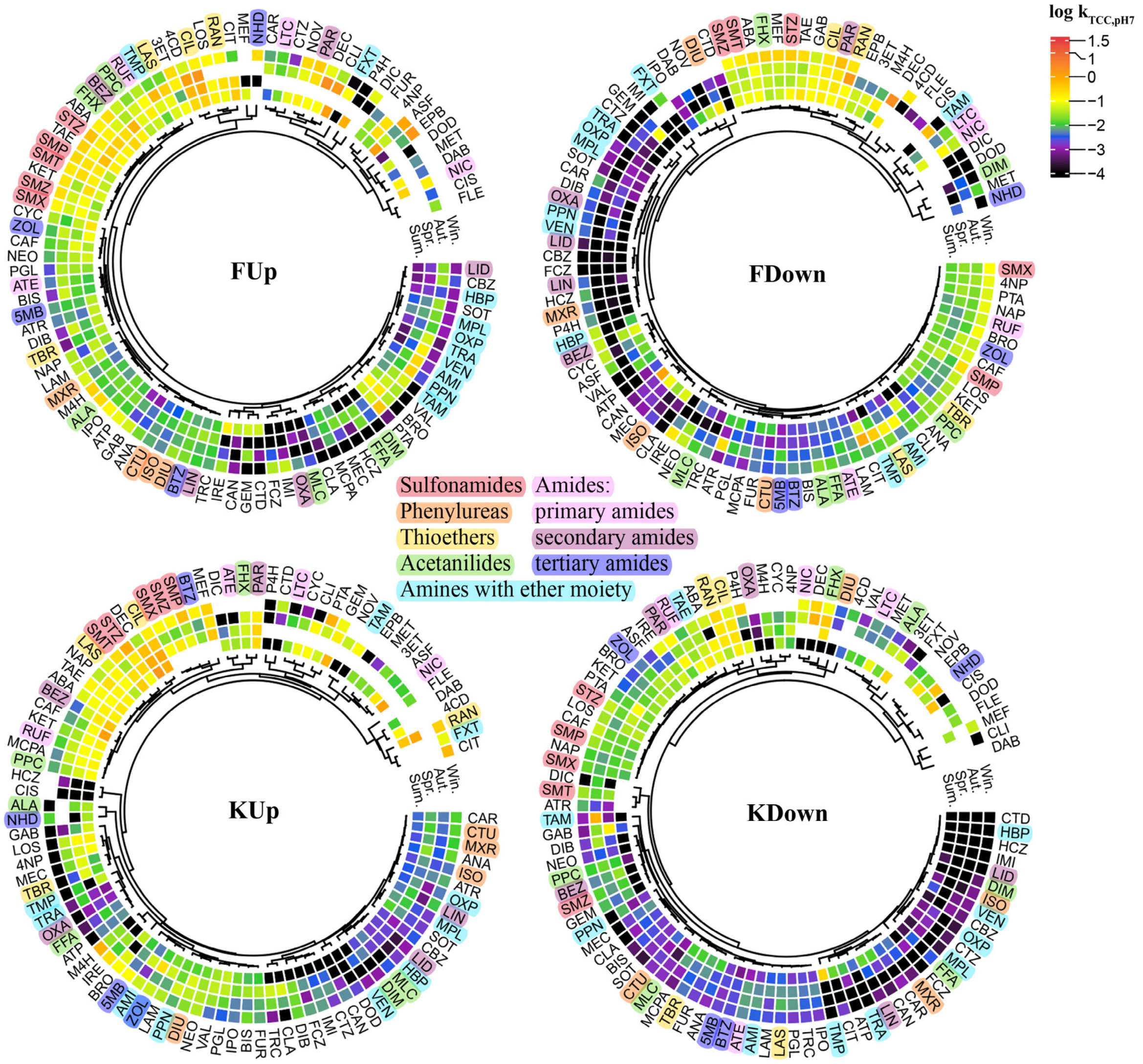

Figure S7. *Clustered heatmaps showing log k (d^{-1}), log k_{pH7} (d^{-1}), and log $k_{TCC,pH7}$ (d^{-1}) of the studied compounds. The chemicals are clustered according to the magnitude and seasonality of the rate constants. All of the estimated k values were used regardless of whether or not they were significantly different from 0. When log k was <-4 or a negative k was not significantly different from 0 (p-value > 0.05), log k was set to -4. From the outer ring to the inner ring, the results are shown in the order of winter (Win.), autumn (Aut.), spring (Spr.), and summer (Sum.). Blank cells indicate that k was not available. For elaboration of the compound name abbreviations see Table S1.*

S6. Significant difference in the seasonality of the rate constants

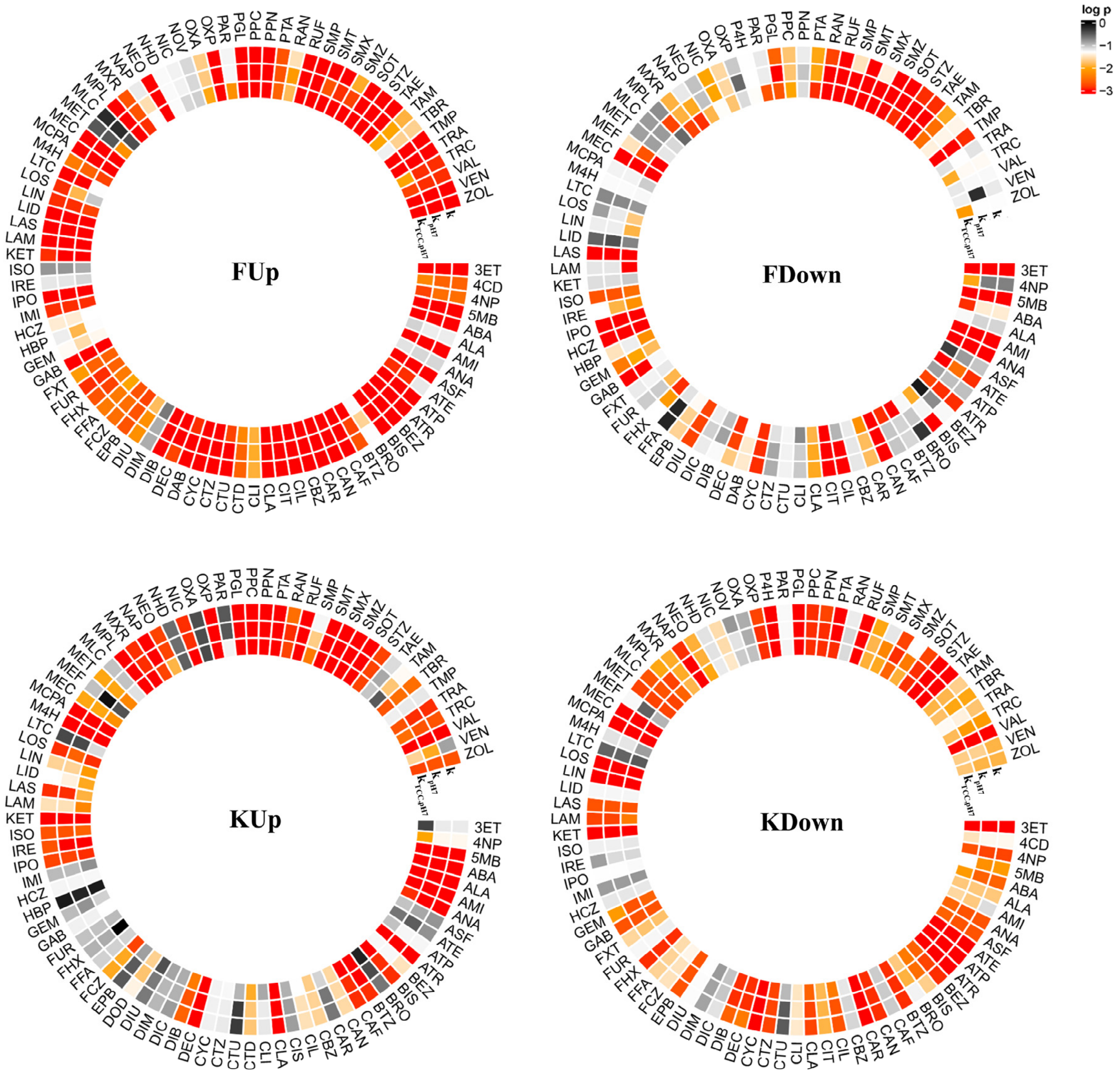

Figure S8. *Heatmaps displaying the significance of the seasonality in k, k_{pH7}, and $k_{TCC,pH7}$, expressed as the log p value. ANOVA was performed for log k, log k_{pH7}, and log $k_{TCC,pH7}$ derived independently from the 3 replicate incubations conducted for each test. All measured k were used, regardless of whether they were significantly different from 0 or not. The white color indicates the p-value was equal to 0.05. For elaboration of the compound name abbreviations see Table S1.*

S7. Arrhenius relationship between k, k_{pH7}, and $k_{TCC,pH7}$ and temperature (T, K).

Table S9. *Arrhenius relationship between chowclassifier generated k, k_{pH7}, and $k_{TCC,pH7}$ and temperature (T, K). R^2 values from the regression of log k, log k_{pH7}, and log $k_{TCC,pH7}$ vs 1/T. Only the compounds that had biodegradation rates significantly different from 0 (p-value > 0.05) in all 4 seasons were compared. N.A. indicates that a given compound didn't have a valid k in at least one season. Cells marked in blue indicate there was a positive correlation between log k, log k_{pH7}, or log $k_{TCC,pH7}$, and 1/T. Text labeled in red indicates R^2 > 0.7. NegCorr.: Percentage of compounds for which log k, log k_{pH7}, or log $k_{TCC,pH7}$ was negatively correlated (R^2 > 0.7) with 1/T. R^2 values marked in italics indicate the chemical has a significant Arrhenius-type relationship and also has an apparent activation energy (Ea) where the 95% confidence interval intersects the recommended ECHA value (65.4 kJ/mol):*

	FUp			FDown			KUp			KDown		
NegCorr.	28%	14%	2%	7%	11%	4%	38%	38%	45%	41%	30%	22%
Abbr.	k	k_{pH7}	$k_{TCC,pH7}$	k	k_{pH7}	$k_{TCC,pH7}$	k	k_{pH7}	$k_{TCC,pH7}$	k	k_{pH7}	$k_{TCC,pH7}$
3ET	0.21	0.21	0.40	N.A.	N.A.	N.A.	N.A.	N.A.	N.A.	N.A.	N.A.	N.A.
4NP	N.A.	N.A.	N.A.	0.70	0.72	0.98	N.A.	N.A.	N.A.	N.A.	N.A.	N.A.
5MB	0.69	0.68	0.54	0.05	0.06	0.41	0.77	0.76	0.57	0.08	0.08	1.87E-04
ABA	0.61	0.61	0.13	0.01	0.01	0.73	0.65	0.65	0.97	N.A.	N.A.	N.A.
ALA	0.59	0.59	0.74	0.44	0.44	0.82	N.A.	N.A.	N.A.	N.A.	N.A.	N.A.
ANA	N.A.	N.A.	N.A.	N.A.	N.A.	N.A.	0.07	0.07	0.08	0.79	0.79	0.69
ATE	0.56	0.50	0.15	0.22	0.16	0.42	N.A.	N.A.	N.A.	0.36	0.21	0.29
ATP	0.38	0.38	0.69	N.A.	N.A.	N.A.	N.A.	N.A.	N.A.	N.A.	N.A.	N.A.
ATR	0.50	0.50	0.35	N.A.	N.A.	N.A.	N.A.	N.A.	N.A.	N.A.	N.A.	N.A.
BEZ	0.89	0.58	0.44	N.A.	N.A.	N.A.	N.A.	N.A.	N.A.	N.A.	N.A.	N.A.
BIS	*0.70*	0.47	0.27	0.25	0.07	0.90	0.17	3.09E-03	3.91E-04	0.96	0.64	0.56
BTZ	0.05	1.41E-03	0.53	3.44E-03	0.05	0.94	N.A.	N.A.	N.A.	0.20	0.21	0.34
CAF	*0.85*	*0.85*	0.68	0.57	0.57	0.94	*0.90*	*0.90*	*0.83*	0.61	0.61	0.77
CAR	N.A.	N.A.	N.A.	N.A.	N.A.	N.A.	0.49	0.49	0.54	N.A.	N.A.	N.A.
CIL	0.44	0.44	0.17	0.99	0.99	0.94	*0.73*	*0.73*	0.94	0.74	0.74	0.67
CTU	0.16	0.16	0.12	N.A.	N.A.	N.A.	0.23	0.23	0.24	0.35	0.35	0.19
CYC	0.43	0.43	0.30	N.A.	N.A.	N.A.	N.A.	N.A.	N.A.	N.A.	N.A.	N.A.

DEC	N.A.	N.A.	N.A.	N.A.	N.A.	N.A.	*0.84*	*0.84*	*0.94*	N.A.	N.A.	N.A.
DIU	0.02	0.02	0.23	N.A.	N.A.	N.A.	3.66E-03	3.66E-03	1.46E-04	N.A.	N.A.	N.A.
FHX	0.54	0.28	0.06	0.53	0.50	0.81	N.A.	N.A.	N.A.	N.A.	N.A.	N.A.
FUR	N.A.	N.A.	N.A.	0.35	0.35	0.01	0.31	0.31	0.31	0.88	0.88	0.90
GAB	0.51	0.51	0.66	0.99	0.99	0.91	N.A.	N.A.	N.A.	N.A.	N.A.	N.A.
HBP	0.87	0.65	0.07	N.A.	N.A.	N.A.	N.A.	N.A.	N.A.	N.A.	N.A.	N.A.
IPO	0.40	0.40	0.71	N.A.	N.A.	N.A.	0.93	0.93	0.78	N.A.	N.A.	N.A.
IRE	N.A.	N.A.	N.A.	N.A.	N.A.	N.A.	N.A.	N.A.	N.A.	3.75E-04	1.13E-03	0.01
ISO	0.32	0.32	3.37E-03	N.A.	N.A.	N.A.	0.01	0.01	0.04	N.A.	N.A.	N.A.
KET	0.72	0.02	0.04	0.59	0.09	0.11	*0.91*	0.99	0.91	0.91	0.98	0.98
LAM	0.12	0.12	0.34	0.34	0.34	0.92	0.01	0.01	2.07E-08	0.30	0.30	0.25
LID	2.60E-03	0.01	0.01	N.A.	N.A.	N.A.	N.A.	N.A.	N.A.	N.A.	N.A.	N.A.
LIN	0.36	0.36	0.04	N.A.	N.A.	N.A.	N.A.	N.A.	N.A.	N.A.	N.A.	N.A.
LOS	N.A.	N.A.	N.A.	N.A.	N.A.	N.A.	N.A.	N.A.	N.A.	0.27	0.11	0.06
M4H	0.64	0.63	0.78	N.A.	N.A.	N.A.	N.A.	N.A.	N.A.	N.A.	N.A.	N.A.
MCPA	N.A.	N.A.	N.A.	N.A.	N.A.	N.A.	*0.82*	*0.82*	*0.95*	*0.92*	*0.92*	*0.93*
MPL	0.92	0.59	0.35	N.A.	N.A.	N.A.	0.94	0.81	0.84	N.A.	N.A.	N.A.
MXR	0.47	0.47	0.71	N.A.	N.A.	N.A.	0.36	0.36	0.35	N.A.	N.A.	N.A.
NAP	N.A.	N.A.	N.A.	0.08	0.50	0.83	N.A.	N.A.	N.A.	0.97	0.35	0.25
NEO	0.99	0.99	0.99	N.A.	N.A.	N.A.	0.18	0.18	0.65	N.A.	N.A.	N.A.
OXP	0.11	0.23	0.04	N.A.	N.A.	N.A.	0.06	0.03	3.40E-03	N.A.	N.A.	N.A.
PAR	N.A.	N.A.	N.A.	0.34	0.35	0.91	N.A.	N.A.	N.A.	0.15	0.15	0.23
PGL	*0.96*	*0.84*	0.68	0.70	*0.71*	0.36	0.93	*0.88*	0.99	0.20	0.10	0.05
PPC	0.85	0.85	0.45	N.A.	N.A.	N.A.	*0.90*	*0.90*	1.00	N.A.	N.A.	N.A.
PTA	N.A.	N.A.	N.A.	0.03	0.03	0.72	N.A.	N.A.	N.A.	0.45	0.45	0.44
RAN	N.A.	N.A.	N.A.	0.03	0.03	0.07	N.A.	N.A.	N.A.	0.56	0.47	0.57
RUF	0.66	0.66	0.21	0.73	0.73	0.92	*0.76*	*0.76*	0.94	0.90	0.90	0.82
SMP	1.29E-04	0.36	0.59	5.44E-04	0.06	0.34	N.A.	N.A.	N.A.	0.48	0.76	0.91
SMT	0.04	0.11	0.41	0.62	0.03	0.24	0.41	0.15	0.05	N.A.	N.A.	N.A.
SMX	0.49	2.49E-03	0.47	0.02	0.41	0.83	N.A.	N.A.	N.A.	0.02	0.05	0.27

SMZ	0.03	0.44	0.70	0.61	0.24	0.39	N.A.	N.A.	N.A.	N.A.	N.A.	N.A.
SOT	0.43	0.32	0.12	N.A.	N.A.	N.A.	0.80	0.99	0.85	0.98	0.85	0.81
STZ	0.33	0.04	0.40	0.05	0.05	0.37	0.31	0.72	0.70	0.01	0.02	0.11
TAE	0.73	0.13	3.94E-03	0.56	0.08	0.03	0.59	0.28	0.08	0.88	0.49	0.42
TBR	N.A.	N.A.	N.A.	0.05	0.05	0.06	N.A.	N.A.	N.A.	0.49	0.49	0.38
TMP	0.04	0.05	4.83E-04	N.A.	N.A.	N.A.	N.A.	N.A.	N.A.	N.A.	N.A.	N.A.
TRA	0.01	0.06	0.01	N.A.	N.A.	N.A.	N.A.	N.A.	N.A.	N.A.	N.A.	N.A.
TRC	0.86	0.86	0.52	N.A.	N.A.	N.A.	N.A.	N.A.	N.A.	N.A.	N.A.	N.A.
VAL	N.A.	N.A.	N.A.	N.A.	N.A.	N.A.	0.59	0.59	*0.79*	N.A.	N.A.	N.A.
ZOL	*0.74*	*0.74*	0.59	0.07	0.06	0.57	9.30E-05	1.21E-04	4.46E-04	0.94	0.94	0.96

S8. Seasonal biodegradation of high-concentration pollutants in rivers

Some of the studied compounds were already present in the water at concentrations > 1 ug L^{-1}, which meant that the microbial communities were exposed to concentrations significantly greater than the spiking concentration, and the exposure concentration varied from season to season (Figure S9). Out of 96 studied compounds, 20, 9, 5, and 2 chemicals were detected at a concentration > 1 µg L^{-1} (spiking concentration) in FDown, KDown, KUp, and FUp, respectively (Figure S9). To better understand the impact of concentration on the seasonality of biodegradation rates while reducing the influence of the neutral fraction and microbial cell counts as confounding factors, the correlation between chemical concentration and $k_{TCC,pH7}$ was explored. At the upstream sites the concentration of the detected compounds was always < 3 µg L^{-1} and there was no correlation ($R^2 < 0.6$) between the concentrations during the different seasons and $k_{TCC,pH7}$. Downstream, no correlation was found for 75% of the compounds, while a positive correlation ($R^2 > 0.6$) was found for 5 of 20 compounds in FDown (p-value > 0.05 for CAF, CAN, and MET, p-value < 0.05 for GEM, PAR) and 2 of 9 compounds in KDown (CAF, CAN, p-value < 0.05).

Caffeine and candesartan were found to have maximum $k_{TCC,pH7}$ at the highest concentrations in both FDown and KDown (Figure S9). Candesartan was detected at the highest concentration and had the maximum $k_{TCC,pH7}$ in summer at both sites. For the other 3 seasons, candesartan biodegraded very slowly and there was no correlation between $k_{TCC,pH7}$ and concentration in the river. Candesartan was not detected upstream of the WWTPs, and it was also not biodegraded in the upstream incubations (Supplemental Dataset S1). Downstream of the WWTPs, caffeine was biodegraded fastest during winter when the caffeine concentration in the river section was highest. However, a correlation between $k_{TCC,pH7}$, and concentrations of caffeine in the river was not observed upstream where caffeine concentrations were always lower than 1.5 µg L^{-1}. Desiante et al. suggested that the biodegradation of caffeine downstream of WWTPs was due to bacteria that had adapted to degrade caffeine due to its high concentrations in the WWTPs and that had been released to the river with WWTP effluent.[7] This could help explain the different biodegradation behaviour of caffeine up- and downstream of WWTPs. Also, rather than a correlation between concentration and $k_{TCC,pH7}$ of candesartan and caffeine, there might be a concentration threshold required to support the degrading organisms. A correlation between $k_{TCC,pH7}$, and concentration in rivers was also found for gemfibrozil, metformin, and paracetamol in FDown (Figure S9). Gemfibrozil and metformin were virtually non-biodegradable when their concentration in the river was low (Figure S9), while $k_{TCC,pH7}$ of paracetamol strongly correlated to its concentration detected in the river ($R^2 = 0.97$). In previous laboratory incubation tests, we also observed a concentration dependence for the biodegradation of caffeine, metformin, and paracetamol.[2] When spiked at 5 ug L^{-1}, the biodegradation rates of those chemicals increased after the third incubation day, indicating a response of the microbial community to the increased concentration level of the specific substrate.

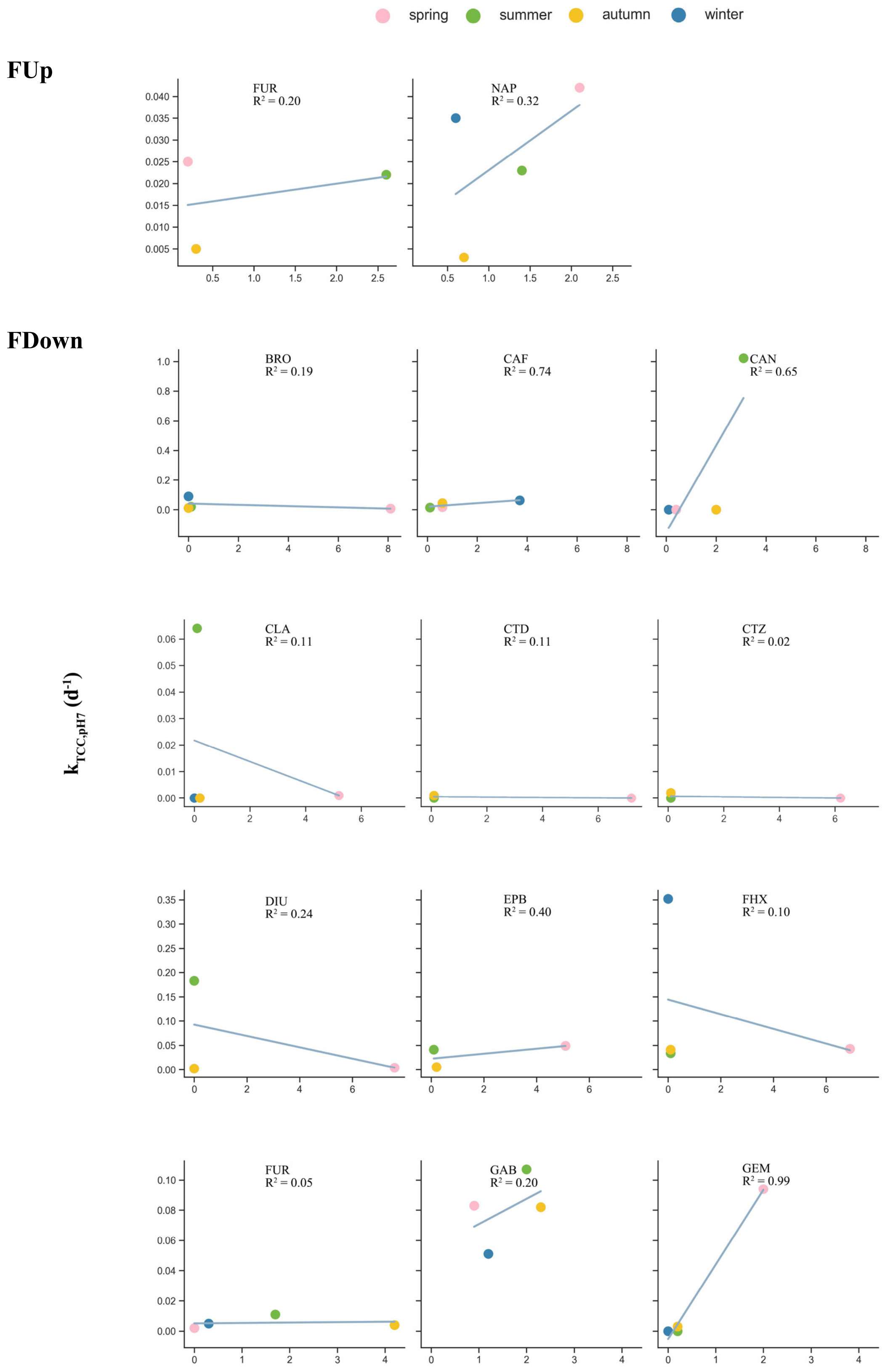
spring summer autumn winter
FUp
FUR $R^2 = 0.20$
NAP $R^2 = 0.32$
FDown
BRO $R^2 = 0.19$
CAF $R^2 = 0.74$
CAN $R^2 = 0.65$
CLA $R^2 = 0.11$
CTD $R^2 = 0.11$
CTZ $R^2 = 0.02$
DIU $R^2 = 0.24$
EPB $R^2 = 0.40$
FHX $R^2 = 0.10$
FUR $R^2 = 0.05$
GAB $R^2 = 0.20$
GEM $R^2 = 0.99$
$k_{TCC,pH7}$ (d^{-1})

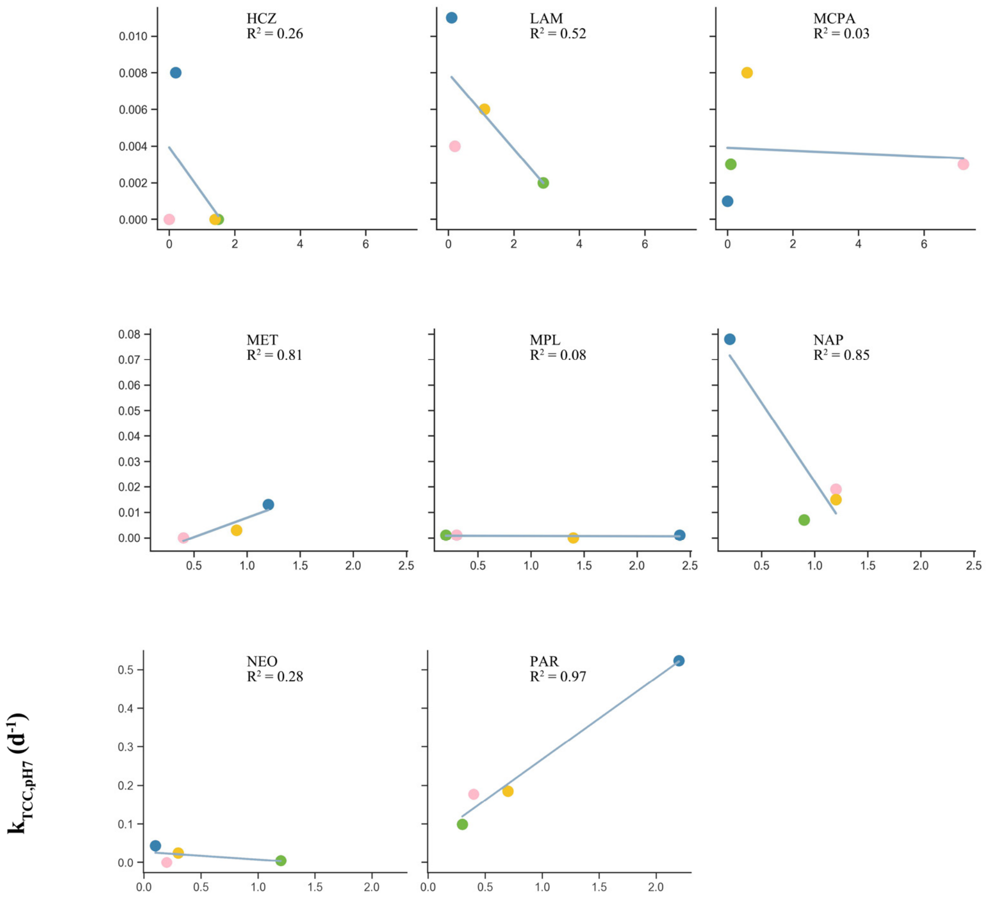
HCZ
R² = 0.26
LAM
R² = 0.52
MCPA
R² = 0.03
MET
R² = 0.81
MPL
R² = 0.08
NAP
R² = 0.85
NEO
R² = 0.28
PAR
R² = 0.97
k TCC,pH7 (d-1)

KUp

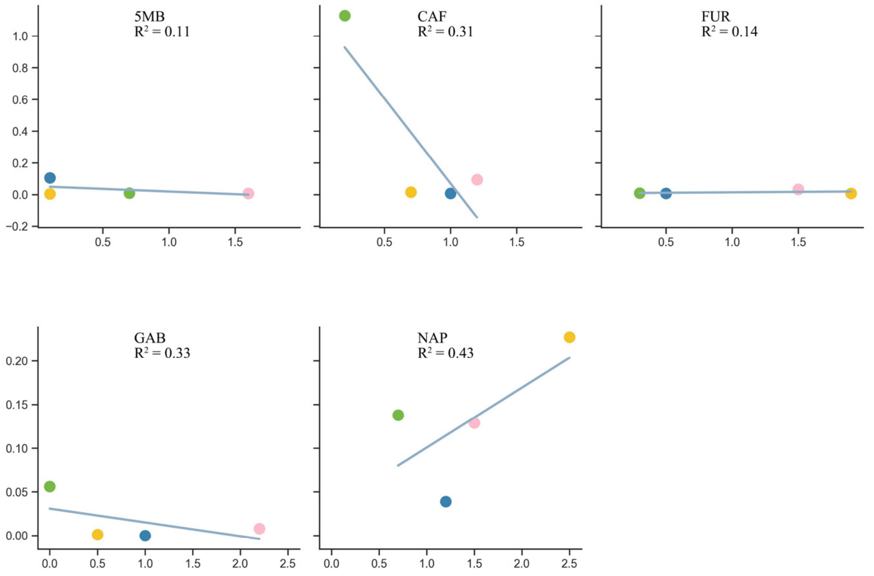
5MB
R² = 0.11
CAF
R² = 0.31
FUR
R² = 0.14
GAB
R² = 0.33
NAP
R² = 0.43

KDown

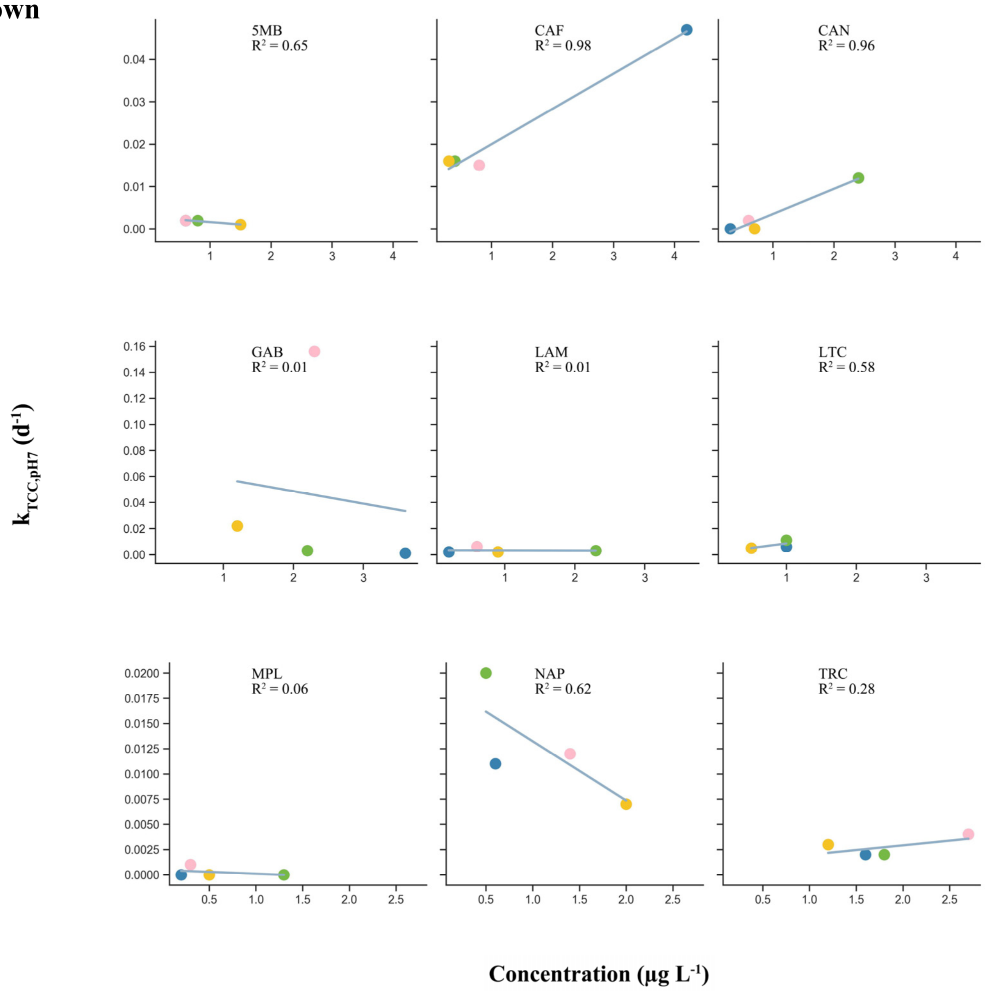

Figure S9. *Correlation between $k_{TCC,pH7}$, and concentration of the compound in the river. The color of the dot indicates the season of the experiment. All the compounds with a concentration in the river > 1 µg L^{-1} were tested for the correlation. For elaboration of compound name abbreviations see Table S1.*

REFERENCES

(1) Seller, C.; Honti, M.; Singer, H.; Fenner, K. Biotransformation of Chemicals in Water–Sediment Suspensions: Influencing Factors and Implications for Persistence Assessment. Environ. Sci. Technol. Lett. 2020, 7 (11), 854–860. https://doi.org/10.1021/acs.estlett.0c00725.

(2) Tian, R.; Posselt, M.; Fenner, K.; McLachlan, M. S. Increasing the Environmental Relevance of Biodegradation Testing by Focusing on Initial Biodegradation Kinetics and Employing Low-Level Spiking. Environ. Sci. Technol. Lett. 2023, 10 (1), 40–45. https://doi.org/10.1021/acs.estlett.2c00811.

(3) Brunius, C.; Shi, L.; Landberg, R. Large-Scale Untargeted LC-MS Metabolomics Data Correction Using between-Batch Feature Alignment and Cluster-Based within-Batch Signal Intensity Drift Correction. Metabolomics 2016, 12 (11), 173. https://doi.org/10.1007/s11306-016-1124-4.

(4) Chow, G. C. Test of Equality Between Sets of Coefficients in Two Linear Regressions. Econometrica 1960, 28 (3), 591–605.

(5) Fisher, F. M. Tests of Equality Between Sets of Coefficients in Two Linear Regressions: An Expository Note. Econometrica2 1970, 38 (2), 361–366.

(6) Schwarzenbach, R. P.; Gschwend, P. M.; Imboden, D. M. Environmental Organic Chemistry, 3rd ed.; John Wiley & Sons: Hoboken, NJ, 2003.

(7) Desiante, W. L.; Carles, L.; Wullschleger, S.; Joss, A.; Stamm, C.; Fenner, K. Wastewater Microorganisms Impact the Micropollutant Biotransformation Potential of Natural Stream Biofilms. Water Research 2022, 217, 118413. https://doi.org/10.1016/j.watres.2022.118413.
